# Seeing around corners with edge-resolved transient imaging

**DOI:** 10.1038/s41467-020-19727-4

**Published:** 2020-11-23

**Authors:** Joshua Rapp, Charles Saunders, Julián Tachella, John Murray-Bruce, Yoann Altmann, Jean-Yves Tourneret, Stephen McLaughlin, Robin M. A. Dawson, Franco N. C. Wong, Vivek K. Goyal

**Affiliations:** 1 Department of Electrical and Computer Engineering, Boston University, 1 Silber Way, Boston, MA 02215 USA; 2 Charles Stark Draper Laboratory, 555 Technology Square, Cambridge, MA 02139 USA; 3 School of Engineering and Physical Sciences, Heriot-Watt University, Edinburgh, EH14 4AS UK; 4 Department of Computer Science and Engineering, University of South Florida, 4202 E. Fowler Avenue, Tampa, FL 33620 USA; 5 INP/ENSEEHIT-IRIT-TeSA, University of Toulouse, Toulouse Cedex 7, Toulouse, 31071 France; 6 Research Laboratory of Electronics, Massachusetts Institute of Technology, 77 Massachusetts Avenue, Cambridge, MA 02139 USA

**Keywords:** Imaging and sensing, Imaging techniques

## Abstract

Non-line-of-sight (NLOS) imaging is a rapidly growing field seeking to form images of objects outside the field of view, with potential applications in autonomous navigation, reconnaissance, and even medical imaging. The critical challenge of NLOS imaging is that diffuse reflections scatter light in all directions, resulting in weak signals and a loss of directional information. To address this problem, we propose a method for seeing around corners that derives angular resolution from vertical edges and longitudinal resolution from the temporal response to a pulsed light source. We introduce an acquisition strategy, scene response model, and reconstruction algorithm that enable the formation of 2.5-dimensional representations—a plan view plus heights—and a 180^∘^ field of view for large-scale scenes. Our experiments demonstrate accurate reconstructions of hidden rooms up to 3 meters in each dimension despite a small scan aperture (1.5-centimeter radius) and only 45 measurement locations.

## Abstract (web summary)

Non-line-of-sight imaging is typically limited by loss of directional information due to diffuse reflections scattering light in all directions. Here, the authors see around corners by using vertical edges and temporal response to pulsed light to obtain angular and longitudinal resolution, respectively.

## Introduction

The ability to see around corners would be profoundly useful in numerous fields, from helping see past partial blockages in medical settings to enabling surveillance while remaining undetected. Among the applications of non-line-of-sight (NLOS) imaging with the most potential is autonomous navigation, which could leverage existing sensing hardware to gather information about hidden pedestrians, vehicles, or other potential obstacles and plan safer trajectories through intersections or into occluded spaces^1^. Practical implementation of anticipatory imaging would require fast acquisition and reconstruction of a large-scale scene with a wide field of view (FOV) in order to detect the most significant obstacles with enough time to react safely. Unfortunately, despite the advances in NLOS imaging over the past decade, current approaches are limited by their long acquisition times, limited FOV, and small scale. In this article, we present a framework for NLOS imaging that scales up the reconstruction volume and FOV from a small number of measurements, presenting a path toward more practical techniques.

The key challenge of NLOS imaging is that optical reflections from diffuse surfaces scatter light in all directions, destroying directional information and diminishing the intensity as the inverse-square of the distance. Diffuse reflections are less problematic for line-of-sight (LOS) imaging (e.g., conventional photography or lidar) because the directionality of light can be preserved through focused illumination or detection, and the radial falloff is often only a problem at large stand-off distances. The difficulty of NLOS imaging arises because of the cascaded diffuse reflections, when light must first reflect off a relay surface before reaching an observer.

Significant strides have been made to counter this challenge over the decade since the conceptualization of imaging beyond the line of sight^2^. The first experimental demonstration of 3D NLOS imaging used active optical illumination, scanning a pulsed laser over a set of points on a relay wall and performing time-resolved sensing to collect light from the same wall^3^. Informative light incurs at least three diffuse reflections in such a scheme: from a visible relay surface to the hidden scene, off the scene, and back from the relay to the detector, so high-resolution transient information is critical to constrain the reconstruction of possible light directions to a more feasible inverse problem. The majority of subsequent work in 3D NLOS imaging followed this basic approach, with some variation in detection configurations and experimental hardware^4–7^.

More recently, the basic imaging configuration has settled to scanning a large 2D grid of points on a planar Lambertian wall, with emphasis on developing ever faster and more accurate reconstruction algorithms, including improved methods of filtering for back-projection^8,9^, fast Fourier transform-based methods such as the light-cone transform^10,11^ and *f*–*k* migration^12^, and various other methods including Fermat paths^13^, Bayesian methods^14^, phasor fields^15,16^, and inverse rendering^17,18^. Regardless of the algorithm used, extremely low levels of informative light for macroscopic scenes typically force active NLOS experiments to use single-photon detectors, acquire transient information from many repeated illuminations, and limit the hidden scene to around 1 meter from the relay surface. Examples that form images of objects at the greatest distances or with the lowest acquisition times increase the laser illumination power to levels that are not eye-safe (e.g., 1 W average optical power at 532 nm)^12,15^, often in combination with retro-reflective hidden objects that reduce radial fall-off.

Beyond pulsed, active illumination, other methods of NLOS imaging have used a wide variety of approaches. A few recent works have demonstrated NLOS images with extraordinarily high resolution without pulsed illumination^19,20^; however, these methods are fundamentally limited to small-scale scenes and thus not practical for applications such as navigation. More prominently, a number of methods that do not capture temporal information have taken advantage of occlusions that block certain light paths^21–32^, similar to coded-aperture imaging or reference structure tomography^33^. For instance, the development of the corner camera^22^ established that the high directional uncertainty created by diffuse reflection from the relay surface can be reduced by an opaque object between the relay surface and the hidden scene. In many cases, exploiting such occlusions enables a completely passive system, using only ambient light to illuminate the hidden scene. For passive NLOS imaging methods, ambient illumination bounces off a hidden scene and a relay surface (usually a visible wall or floor) before reaching a detector. These passive, intensity-only methods generally have less radial fall-off but more uncertainty due to uninformative ambient light, so reconstructions are typically limited to 2D images of table-top scenes^26^ or 1D traces showing objects’ angular positions with respect to a vertical edge^22,27^. Finally, some NLOS imaging approaches aim to avoid diffuse reflections entirely by using modalities (e.g., thermal^34^, acoustic^35^, or radar^36^) operating at long wavelengths, at which optically-rough surfaces appear smooth. While the directionality and signal strength are preserved through one or more specular reflections, such methods measure physical properties other than the optical reflectance, the resolution is lower due to the longer wavelengths, and the specular reflections can lead to confusion in distinguishing between direct and indirect reflections.

Although impressive and promising, most existing NLOS imaging methods are ill-suited to practical deployment. For high-quality reconstructions, active methods that can capture macroscopic scenes require the scanning area to be larger than the orthographic projection of the hidden scene^9^, so a (3 m)^3^ hidden volume would require at least a (3 m)^2^ aperture. Such large, planar relay walls with uniform albedos and bidirectional reflectance distribution functions (BRDFs) do not typically exist in the wild. Furthermore, the large number of scanned illumination points limits the acquisition speed, especially since high-resolution, time-resolved focal plane arrays are not in widespread deployment. Only a handful of active NLOS methods exist that do not require large scanning apertures, but these either require motion of a hidden object^6,37^ or make the strong assumption that the hidden space is an empty polyhedron^7^. Passive methods like the corner camera are promising, in that they use only the visible source of occlusion to resolve details about the hidden space without requiring a separate relay wall^22,27,32^. However, those methods are sensitive to the ambient illumination—requiring the hidden scene to be well-lit, but without the light in the visible scene washing out the measured penumbrae—and are extremely limited in their ability to make inferences about the 3D structure of scenes.

In this work, we propose an active NLOS imaging approach that moves toward more practical sensing scenarios. Our method recovers large-scale NLOS images with a large FOV by combining the small aperture and opportunistic use of visible occluders from passive corner cameras with the precise distance measurement ability of time-resolved active systems into a novel acquisition configuration. We call our method edge-resolved transient imaging (ERTI) because we take advantage of occlusions from vertical edges, in addition to using single-photon-sensitive, time-resolved acquisition. Our method introduces a dual configuration to existing corner cameras by scanning a light source along an arc on the ground plane around a vertical edge, thereby controlling which portion of the hidden space is illuminated, and detecting light from a single spot. Combining pulsed illumination and time-resolved, single-photon sensing yields measurements of the transient response of the illuminated scene. Because of the novel acquisition geometry, differences between histograms of photon detection times from adjacent illumination spots localize the transient response to that of a hemispherical wedge. Light propagation modeling leads to closed-form expressions for the temporal response functions of planar facets extending from the ground plane for a given distance, height, orientation, and albedo. Bayesian inference employing a tailored Markov chain Monte Carlo (MCMC) sampling approach reconstructs planar facets for each wedge and accounts for prior beliefs about the structure of likely scenes. The small number of measurement positions on a small scanning aperture has the potential to enable fast acquisition in more general environments. While not recovering a full 3D image due to reduced resolution in the vertical dimension, ERTI does reconstruct object positioning in the hidden space that would be useful for advanced collision avoidance.

## Results

### Acquisition methodology

Vertical edges, such as those in door frames or at the boundaries of buildings, are ubiquitous and have proven useful for passive NLOS imaging^22,27,32^. In corner cameras, a conventional camera images the ground plane where it intersects with the vertical edge of a wall separating visible and hidden scenes. Whereas light from any visible part of the scene can reach the camera’s FOV, Bouman et al.^22^ showed that the vertical edge occludes light from the hidden scene from reaching certain pixels, depending on their position relative to the vertical edge. This work introduces two changes to how vertical edges enable NLOS imaging. First, rather than using global illumination and spatially-resolved detection, we propose a Helmholtz reciprocal dual of the corner camera, in which the illumination is scanned along an arc centered at the edge, and a bucket detector aimed beyond the edge collects light from both the visible and hidden scenes. Second, we use a pulsed laser and single-photon-sensitive, time-resolved detection instead of a conventional camera.

**Fig. 1:**
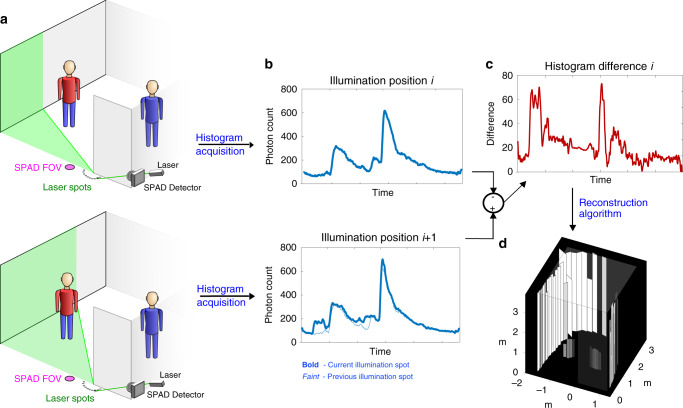
Edge-resolved transient imaging scenario and procedure. **a** Positions along an arc centered on the occluding wall edge are illuminated sequentially by a pulsed laser. **b** A histogram of photon counts over time is collected for each laser illumination spot. The measured histograms contain photons reflected from both the hidden scene and the visible side. **c** Taking differences between sequential histograms on average yields returns originating only from a small wedge within the hidden scene. **d** A hidden area reconstruction using the collection of histogram differences.

The ERTI acquisition methodology is illustrated in Fig. 1. A 532-nm laser at 120 mW average optical power sequentially illuminates 45 spots evenly spaced in angle *θ* from 0 to *π* radians along a semicircle of radius 1.5 cm centered around the edge of the wall. The laser light illuminates an increasing fraction of the hidden scene as the illumination spot moves along the arc toward the hidden area. Each spot *i* is repeatedly illuminated with picosecond-duration pulses at 20-MHz repetition rate for a preset dwell time. Light from each pulse bounces off the Lambertian ground plane and scatters in all directions, reflecting from surfaces in both visible and hidden portions of the scene. A single-photon avalanche diode (SPAD) detector is focused at a small spot roughly 20 cm beyond the vertical edge, enabling collection of light from the entire hidden scene for each illumination spot. After each pulse, a time-correlated single photon counting (TCSPC) module connected to the SPAD records photon detection times with 16-ps resolution, forming a histogram **m**_*i*_ of those photons reflected back to the SPAD. To prevent the direct reflection from overwhelming the much weaker light from the hidden scene, temporal gating is implemented to turn on 3 ns after the direct reflection from the ground reaches the SPAD.

The detected light intensity for spot *i* includes contributions **h**_*i*_ from the hidden scene, **v**_*i*_ from the visible scene, and **b** from the background (a combination of ambient light and dark counts). The background is assumed to have constant intensity over the duration of the acquisition, and because the illumination arc radius is small, the visible scene contribution is approximately constant over all spots, i.e., **v**_*i*_ ≈ **v**_*j*_ for all *i*, *j*. However, illuminating sequentially along an arc will change the parts of the hidden scene that are illuminated. More precisely, a larger area of the hidden scene is illuminated as *i* increases, so **h**_*i*+1_ = **h**_*i*_ + **u**_*i*_, where **u**_*i*_ is the component of the histogram contributed by the portion of the scene illuminated from spot *i* + 1 but not from spot *i*, and **u**_0_ = **0** because only the visible scene is illuminated from the first laser spot. The key idea behind ERTI is that this new contribution can be isolated—thereby regaining NLOS directionality—by considering the difference between successive histograms, that is,

1 $${{\bf{y}}}_{i}={{\bf{m}}}_{i+1}-{{\bf{m}}}_{i}\approx \left({{\bf{v}}}_{i+1}+{\bf{b}}+\sum_{j = 0}^{i}{{\bf{u}}}_{j}\right)-\left({{\bf{v}}}_{i}+{\bf{b}}+\sum_{j = 0}^{i-1}{{\bf{u}}}_{j}\right) \approx\, {{\bf{u}}}_{i}.$$

Due to the hemispherical reflection of light from a Lambertian ground plane and the occlusion effect of the vertical edge, the histogram differences ${\{{{\bf{y}}}_{i}\}}_{i = 1,\ldots ,44}$ correspond to distinct wedges fanned out from the vertical edge. We note that each photon detection time histogram **m**_*i*_ has Poisson-distributed entries, so each histogram difference **y**_*i*_ has entries following the Poisson-difference or Skellam distribution^38^. Moreover, the entries of **y**_*i*_ are conditionally independent given the scene configuration. Note that although the mean visible scene and ambient light contributions are removed by this procedure, they do still contribute to the variance of the observation noise; see Supplementary Note 2, which discusses how working with **y**_*i*_ directly instead of **m**_*i*_ leads to a more efficient reconstruction procedure.

### Light transport model

With access to a large relay wall, most active NLOS methods can recover 3D information from two scan dimensions plus time. Passive reconstructions are more limited, and the vertical edge provides corner cameras with only one dimension of angular resolution^22,27^; coarse range estimates may be recovered from subtle changes in radial falloff patterns for small-scale scenes^32^ or from a stereo pair of corner cameras^22^. ERTI similarly acquires azimuthal resolution from the vertical edge, and the temporal resolution adds a second dimension of depth in the hidden scene. There is no similar mechanism for distinguishing elevation angle: within the wedge formed by measurements at angles *θ*_*i*_ and *θ*_*i*+1_, a point reflector near the ground or floating in air at the same radial distance from the corner would have an identical transient response, neglecting variations due to the view angle relative to the ground.

To solve this identifiability issue, we make two key assumptions about the hidden scenes we expect to encounter. First, we assume that the surfaces of hidden-scene objects that are visible from the base of the vertical edge are vertical and extend upward from the ground plane. While omitting cantilevered or hanging objects like chandeliers, in a world governed by the force of gravity, this approximation covers a large class of objects in both indoor and outdoor scenes, including walls, doors, furniture, humans, buildings, etc. These are also the most important obstacles to identify for robotics or other autonomous navigation. Second, the front surface of an object is approximated as a planar facet that spans the full width of the wedge. While this neglects the curvature of objects such as cylindrical columns, their general shape can be recovered with sufficient azimuthal resolution. For indoor scenes, we also model the optional presence of a ceiling as a single additional surface, assumed parallel to the ground. Despite being a major exception to our vertical-facet model, inclusion of the ceiling component is necessary as it often reflects a significant amount of light.

Together, these assumptions lead to a middle-ground 2.5D representation of the scene as a collection of planar facets, augmenting a 2D plan view (the positions and orientations of surfaces in the hidden space) with the height of each surface. Within a wedge, a planar facet requires only four parameters. The time of flight to the surface naturally yields the radial distance, denoted *ρ*. Relying on the so-called gravity prior, the shape of the transient then contains information about the facet height *η* and orientation angle *ϕ*, with the amplitude also determined by the surface albedo *α*. These parameters are defined more precisely later in the text.

We now highlight key aspects of our facet response derivation. Given that the illumination arc radius, the SPAD FOV, and their separation are all small, the acquisition configuration is approximately confocal, with illumination and detection occurring at a single point at the base of the vertical edge^10^. Without loss of generality and to simplify the light transport model, this point is designated as the origin of the coordinate system both spatially and temporally. The additional light travel time from the laser and back to the detector are subtracted away.

The transient light transport for NLOS imaging with pulsed laser illumination and a focused detector is intricate but can be well approximated by factors accounting for the round-trip time of flight, radial falloff, and cosine-based corrections for Lambertian reflection^4,23^. With *α*(**p**) denoting the albedo of a point **p** on a hidden surface ${\mathcal{S}}$, the transient light transport describing the photon flux at time *t* is given as

2 $$L(t)=\int_{{\mathcal{S}}}\alpha ({\bf{p}})\frac{G({\bf{p}})}{\Vert {\bf{p}}{\Vert }^{4}}\,\delta(2\Vert {\bf{p}}\Vert /c-t)\ d{\bf{p}},$$

where the BRDF factor is defined as

3 $$G({\bf{p}})=\,\cos\left(\measuredangle ({\bf{p}},{{\bf{n}}}_{{\bf{f}}})\right)\, \cos(\measuredangle (-{\bf{p}},{{\bf{n}}}_{{\bf{p}}}))\\ \, \cos(\measuredangle (-{\bf{p}},{{\bf{n}}}_{{\bf{p}}})) \, \cos\left(\measuredangle ({\bf{p}},{{\bf{n}}}_{{\bf{f}}})\right),$$

**Fig. 2:**
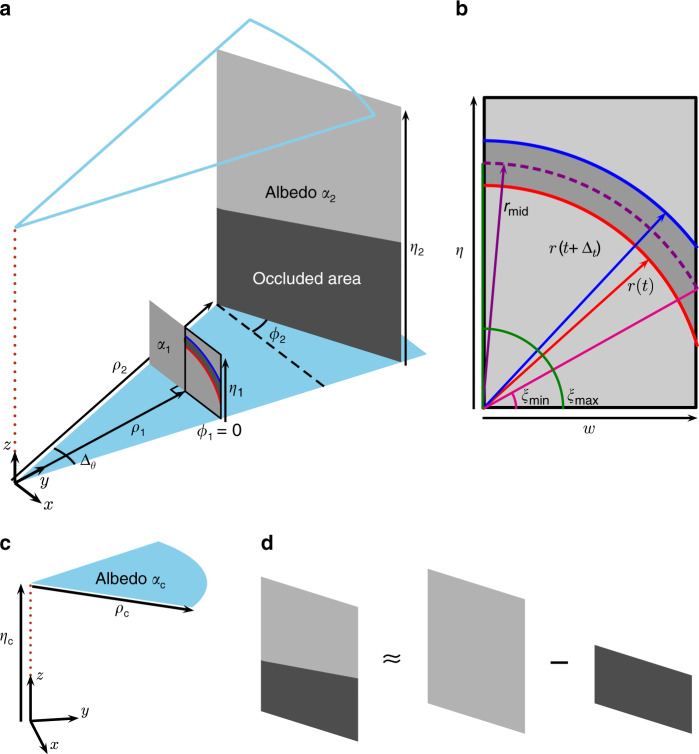
Planar facet scene representation. **a** The contents of a wedge spanning angle *Δ*_*θ*_ are represented by a set of planar facets parameterized by a distance *ρ*, height *η*, albedo *α*, and orientation angle *ϕ*. **b** The basic transient light transport is computed for the region illuminated between times *t* and *t* + *Δ*_*t*_ of one-half of a fronto-parallel facet. For facets with nonzero *ϕ*, the full response linearly combines two half-facet responses with the distance and widths adjusted according to orientation angle. **c** The response from a ceiling component is computed similarly to a fronto-parallel facet. **d** Only the portion of a facet not occluded by a closer facet contributes to the total response. The effects of varying the facet parameters on the facet transient response are visualized in Supplementary Movie 1.

**n**_**p**_ is the unit surface normal at **p**, and **n**_**f**_ = [0, 0, 1] is the unit normal of the ground plane. Because histogram differences isolate the response from a single wedge, we can compute the response for each wedge of the hidden scene independently. As shown in Fig. 2a, the wedge formed between illumination angles *θ*_*i*+1_ and *θ*_*i*_ is defined to have angular width *Δ*_*θ*_ = *θ*_*i*+1_ − *θ*_*i*_.

We start by considering a fronto-parallel half-facet as outlined in bold in Fig. 2a and highlighted in Fig. 2b. Suppose the half-facet has width *w*, height *η*, and uniform albedo *α*, and is located a distance *d* along the *y*-axis (without loss of generality, since the coordinate system can be chosen arbitrarily for each wedge) with surface normal **n**_**p**_ = [0, −1, 0]. The transient response from the half-facet is then given as

4 $$h(t;\alpha ,d,w,\eta )= \, \alpha {d}^{2}\int_{0}^{w}\int_{0}^{\eta }\frac{{z}^{2}}{{({x}^{2}+{d}^{2}+{z}^{2})}^{4}}\\ \, \delta\left(\frac{2}{c}\sqrt{{x}^{2}+{d}^{2}+{z}^{2}}-t\right)\ dz\ dx.$$

We account for the temporal discretization of TCSPC systems, which accumulates photon flux over time bins [*t*, *t* + *Δ*_*t*_]. Using the confocal approximation, objects with the same path length to the origin lie on a sphere, so over one time bin, the intersection of the sphere with a planar facet is a section of a circular annulus^39^. Solving Eqn. (4) is thus more natural in cylindrical coordinates (*r*, *ξ*, *y*), where *r*^2^ = *x*^2^ + *z*^2^, $z=r\sin \xi$, $x=r\cos \xi$, and  *dz* *dx* = *r* *dr* *dξ*. For light with round-trip travel time *t*, the radius of a circular section of a fronto-parallel facet is $r(t)=\sqrt{{\left(tc/2\right)}^{2}-{d}^{2}}$. Hence, Eqn. (4) integrated over one time bin reduces to

5 $${h}_{t}(\alpha ,d,w,\eta ,{\Delta }_{t})\approx \alpha {d}^{2}\int_{r(t)}^{r(t+{\Delta }_{t})}\frac{{p}^{3}}{{({p}^{2}+{d}^{2})}^{4}}\ dp \int_{{\xi }_{\min }}^{{\xi }_{\max }}{\sin }^{2}\xi \ d\xi$$

for the range of valid times $t\in [2d/c,2\sqrt{{d}^{2}+{\eta }^{2}+{w}^{2}}/c]$. The angular limits depend on *r*, but since the temporal discretization for TCSPC systems is fine, a close approximation uses ${\xi }_{\min }={\cos }^{-1}\left[\min \{1,w/{r}_{{\rm{mid}}}\}\right]$ and ${\xi }_{\max }={\sin }^{-1}\left[\min \{1,\eta /{r}_{{\rm{mid}}}\}\right]$, which are defined with respect to the middle radius of the annulus ${r}_{{\rm{mid}}}=\left[r(t)+r(t+{\Delta }_{t})\right]/2$ and the half-facet width $w=d\tan ({\Delta }_{\theta }/2)$, as shown in Fig. 2b. Finally, each integral in Eqn. (5) has a closed-form solution, so the half-facet transient response can be easily computed for a valid time bin as

6 $${h}_{t}(\alpha ,d,w,\eta ,{\Delta }_{t}) \approx \, \frac{\alpha {d}^{2}}{24}[{\xi }_{\max }-{\xi }_{\min }+\sin ({\xi }_{\min })\cos ({\xi }_{\min })\\ \, -\sin ({\xi }_{\max })\cos ({\xi }_{\max })]\\ \, \times \left[\frac{3{r}^{2}(t)+{d}^{2}}{{({r}^{2}(t)+{d}^{2})}^{3}}-\frac{3{r}^{2}(t+{\Delta }_{t})+{d}^{2}}{{({r}^{2}(t+{\Delta }_{t})+{d}^{2})}^{3}}\right].$$

The full response of a fronto-parallel facet simply sets *d* = *ρ* and doubles the basic half-facet response due to symmetry. The full response for a more general facet orientation angle *ϕ* ≠ 0 adjusts the distance parameter based on the rotation angle and linearly combines two half-facet responses with different widths (see Supplementary Note 1), and is likewise efficient to compute. The transient response from an entire wedge also incorporates the portion of the ceiling within that wedge (Fig. 2c), which has a closed-form approximation similar to that of a fronto-parallel facet. Finally, if multiple facets appear within a wedge, the total wedge response non-linearly combines facet contributions by removing the response components from more distant facets that are occluded by closer facets (Fig. 2d). The full derivation of the transient response for a wedge can be found in Supplementary Note 1.

### Reconstruction approach

**Fig. 3:**
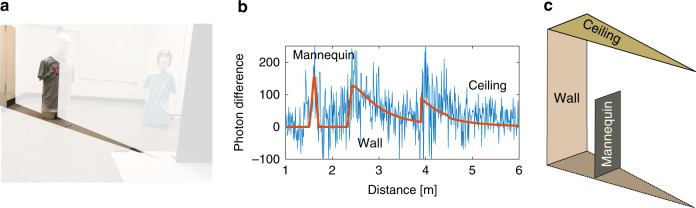
Reconstruction of a hidden wedge. **a** A photograph of an example hidden scene, highlighting the wedge to be reconstructed. **b** The proposed algorithm fits the planar facet model (orange) to the acquired histogram difference (blue), identifying contributions from the mannequin, wall, and ceiling. The time to each surface yields the position of the facets, the response shape provides information about the height and orientation, and the amplitude of the response is proportional to the surface albedo. **c** This information is used to form a 2.5 D reconstruction of the hidden wedge.

The reconstruction algorithm aims to fit the planar facet model to the observed histogram differences, as illustrated in Fig. 3.

One major difficulty of NLOS imaging is that the number of surfaces per resolved wedge is unknown a priori and can vary across the hidden scene. Some wedges have only ceiling and wall contributions, whereas other wedges contain additional objects, such as the mannequins in our experiments. We simultaneously process all histogram differences to capture spatial dependencies between facet configurations across wedges of the hidden scene.

The performance of our reconstruction method relies on a carefully tailored scene model, which must be both flexible and informative while remaining computationally tractable. In natural hidden scenes, we observe that facets tend to be spatially clustered, with clusters representing different objects in the room. We also observe that the positions of facets belonging to the same object tend to describe a 1D manifold. For example, the walls of the room can be described by a concatenation of facets forming the perimeter of the hidden scene. Moreover, the parameters of neighboring facets belonging to the same object are strongly correlated. For example, wall facets tend to share similar heights, albedos and orientations.

These assumptions about scene structure are incorporated into the model via a Bayesian framework by assigning a prior distribution *p*(Φ, ***χ***) to the set of facets Φ (distance, height, albedo, and orientation angle) and ceiling parameters ***χ*** (height and albedo). Interpreting the positions of the planar facets as points in 2D space (top view of the hidden room), we define a spatial point process prior model that favors structured configurations (clusters of 1D manifolds). This model is inspired by recent 3D reconstruction algorithms for LOS single-photon lidar that represent surfaces as 2D manifolds^40,41^. Inference about the most likely room configuration (Φ, ***χ***) is carried out by maximizing the posterior distribution

7 $$p(\Phi ,{\boldsymbol{\chi }}\ | \ {\{{{\bf{y}}}_{i}\}}_{i})\propto p({\{{{\bf{y}}}_{i}\}}_{i}\ | \ \Phi ,{\boldsymbol{\chi }}) \, p(\Phi ,{\boldsymbol{\chi }}),$$

where $p({\{{{\bf{y}}}_{i}\}}_{i}\ | \ \Phi ,{\boldsymbol{\chi }})$ denotes the likelihood of observing the measurements given the room parameters (Φ, ***χ***), which is computed using the light transport model defined in the previous section.

To solve this problem, we develop a reversible-jump MCMC algorithm^42^ that can estimate both the number of facets and their respective parameters. At each iteration, the algorithm proposes a random, yet guided, modification to the configuration of facets (e.g., addition or removal of a facet), which is accepted with a pre-defined rule (the Green ratio^42^). Note that this approach only requires the local evaluation of the forward model, i.e., for individual wedges, which takes advantage of the fast calculations based on Eqn. (6). In particular, we can efficiently take into account non-linear contributions due to occlusions between facets of a given wedge. By designing tailored updates (see Supplementary Note 2), the algorithm finds a good fit in few iterations, resulting in execution times of approximately 100 s, which is less than the acquisition time of the system.

### Limitations of conventional methods

Conventional active NLOS imaging methods that scan a relay wall can recover three-dimensional details of a hidden scene with surprising accuracy. However, these methods require a large virtual aperture (the area scanned by the laser and/or detector) and a large number of measurement points. The lateral resolution depends on the sampling density and inversely upon the virtual aperture size^10,12,15^, which is thus preferred to be as large as possible. The size of the recovered volume also depends on the aperture size: finite scan apertures are sufficient so long as the orthographic projection of the hidden scene is smaller than—and contained within—the scan area^9^. Conventional methods are thus ill-suited to recover large volumes, especially if only a small scan area is available or if the number of measurements is limited for the purpose of acquisition speed.

**Fig. 4:**
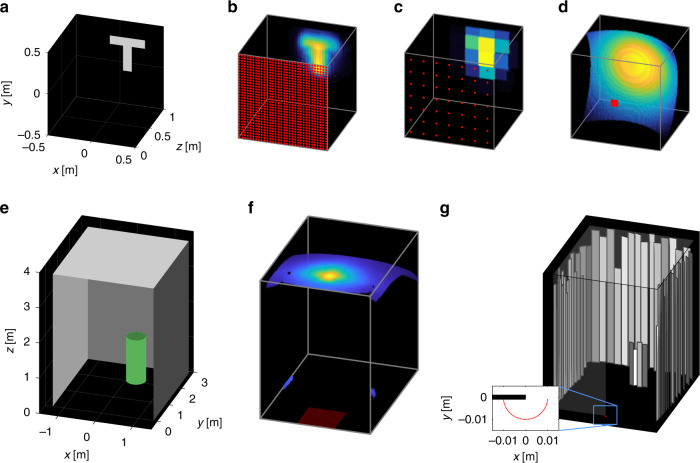
Conventional NLOS limitations. Simulations of confocal measurements for a planar, T-shaped object (**a**) highlight the resolution and field-of-view limitations of conventional NLOS methods. Laser illumination positions are shown as red dots throughout. Reconstructions using the *f*–*k* migration algorithm^12^ (**b**–**d**) show degradation as the aperture sizes and numbers of measurement points decrease. Hence, large-scale scenes (**e**) yield poor reconstructions (**f**), even from an aperture size of (1 m)^2^. By taking advantage of existing vertical edges (**g**), ERTI is still able to accurately reconstruct large scenes despite a small aperture and few measurements.

  Fig. 4 highlights how these limitations affect reconstruction quality. The top row shows reconstructions from simulated confocal measurements of the planar, T-shaped object in Fig. 4a. We use the *f*–*k* migration algorithm^12^ for all comparisons because it has similar computational efficiency to the light-cone transform^10^ but typically yields sharper results and fewer streak-like artifacts without regularization. The reconstruction in Fig. 4b is fairly accurate, since the scan aperture (1 m × 1 m, shown in the inset) and number of measurement points (32 × 32) is sufficiently large, and the hidden object’s projection falls within the scan area. Using a smaller number of measurements (7 × 7) reduces the possible reconstruction resolution in Fig. 4c, so the T shape is barely resolved. Recovering a 1 m × 1 m × 1 m volume from a smaller aperture size (3 cm × 3 cm) requires first laterally padding the transient measurements with zeros. Although the depth is correctly estimated in Fig. 4d, the T shape is completely blurred in the transverse direction, showing the difficulty of using an aperture size smaller than the object projection.

These results highlight why conventional methods are not practical for imaging large-scale scenes, especially without a large relay wall. The same 1 m × 1 m scan aperture with 32 × 32 illumination spots that succeeds for the small object in Fig. 4b is not sufficient when placed on the floor at the entrance of a room-scale scene as shown in Fig. 4e. Conventional methods can recover only the ceiling directly opposite the scan aperture, but the walls and cylinder outside the field of view are completely blurred in Fig. 4f. Taking advantage of existing vertical-edge occluders enables ERTI to avoid the limits of conventional methods in reconstructing scenes far larger than the scan aperture. Despite a smaller aperture (1-cm radius arc) and the same number of measurement points (49) as the failed conventional reconstruction in Fig. 4d, the ERTI reconstruction in Fig. 4g accurately positions the ceiling, walls, and visible components of the cylinder, even accounting for occlusions between objects.

### Experimental reconstructions

Our reconstruction approach is assessed using measurements of challenging indoor scenes containing multiple objects with a variety of depths, heights, rotation angles, and albedos. The hidden scenes consist of an existing room structure modified by movable foamcore walls, with several objects placed within the scene. Black foamboard is used to create a vertical edge with reduced initial laser reflection intensity. Due to the specular nature of the existing floor, foamcore coated with a flat, white spray paint is used to achieve a more Lambertian illumination and detection relay surface, enabling even light distribution to all angles of the hidden scene.

**Fig. 5:**
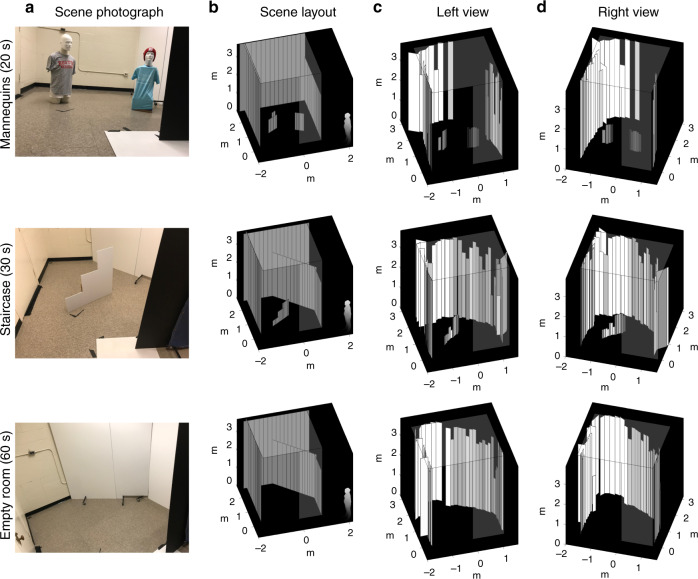
ERTI reconstructions of hidden scenes. **a** Photographs show hidden rooms constructed from existing walls and foamcore panels. The rooms contain either two mannequins or a planar staircase or are empty. **b** Diagrams show the approximate layout of the hidden scene plus an observer at the position of the acquisition equipment. **c** Left & **d** right views of the reconstructed scenes, with the estimated ceiling height and known occluder edge shown partially transparent for context. Representative results are shown for different acquisition durations per illumination spot. Shorter acquisition times are sufficient for correctly localizing the foreground objects with height estimates within  ≈ 10 cm, whereas longer times allow for more accurate estimation of distant facet heights and orientations. See Supplementary Note 6 for the effects of varying the acquisition time on the reconstructions.

Fig. 5 shows multiple views of the results of our reconstruction method for three example scenes. Each dataset was acquired from 45 illumination positions, with acquisition times of 20 s per illuminated spot for the mannequins, 30 s per illuminated spot for the staircase, and 60 s per illuminated spot for the empty room. The approximate scene layout is displayed for reference using measurements from a laser distance meter. The foreground objects, the ceiling height, and most of the wall components are recovered, with visual inspection confirming approximately correct positions and orientations. The planar staircase object is useful as a height resolution test, with the average facet height for the 30-, 60-, and 90-cm steps measured to be 41.1, 54.3, and 92.1 cm, respectively, yielding roughly 10-cm accuracy. The most challenging components to accurately recover are wall facets that are occluded, oblique-angled, and/or far from the vertical edge. Additional simulated and experimental results varying the scene content, acquisition duration, and number of illumination spots are presented in the Supplementary Note 6, showing in part how the reconstruction algorithm performs when the scene content does not match the modeling assumptions.

In general, the histogram differences from real experimental data with reasonably short acquisition times are extremely noisy (see Fig. 3), which makes accurate estimation challenging. Situations in which the visible scene response is large, or there is significant ambient background light, result in high variance in the measurements. Furthermore, the variance in the measurements due to the hidden scene itself increases linearly as a function of the illumination angle *θ*, making the estimation more difficult at higher angles. Despite these effects, our reconstruction approach is quite robust to low signal strength and a high number of background counts, as confirmed by additional simulations presented in the Supplementary Note 7.

## Discussion

We have presented a method for imaging large-scale scenes outside the line of sight by measuring the transient light transport from scene illumination constrained by a visible occluder. Other time-resolved methods for NLOS imaging using a relay wall have ellipsoidal uncertainty in the position of a reflecting surface, requiring a large scan area with many illumination and/or detection points. The edge-resolving property of ERTI combined with the histogram differencing reduces the uncertainty from two dimensions to one, requiring dramatically fewer distinct measurements (e.g., 45 illumination locations) and a smaller aperture (e.g., 1.5 cm arc) than previous methods, as well as simplifying reconstruction. Moreover, existing methods using the floor as a relay surface depend on differences between multiple acquisitions to isolate 2D positions of moving objects from the clutter reflections from static surfaces^6^, whereas ERTI recovers the entire static scene.

The use of a planar facet-based representation results in a number of key advantages over other active NLOS methods, which typically describe the scene as a volume of isotropically reflecting voxels. The larger surface area of a facet relative to a voxel results in a stronger transient, which makes matching facet parameters easier. ERTI can thus image large-scale scenes despite the strong attenuation due to radial falloff and without engineering the BRDF of the scene, e.g., using retro-reflectors to return more light. A surface-based representation is also more memory-efficient than voxelization, which scales cubically with the scene dimension. Finally, our scene representation makes it possible to easily incorporate nonlinear transport effects into the model, since it is straightforward to calculate the projection of a near facet onto a more distant one to determine the contribution from the facet that would be masked by occlusion. Volumetric methods seldom treat occlusions within the scene due to this nonlinearity, and existing solvers are extremely slow, requiring several hours despite substantial computational resources^43^.

To our knowledge, only one previous paper tried to likewise reconstruct a scene represented by a collection of planes^7^. That paper claimed the transient response from a plane could not be computed in closed form and thus resorted to forming a dictionary of responses from Monte Carlo simulation with various sets of parameters. The shape of an empty room was then recovered by fitting the measured response with a sparse set of dictionary elements. By taking advantage of the vertical edge, ERTI allows a far more flexible approach. As we showed, the transient response from a planar facet can be analytically derived from first principles, with approximations that are minor due to the fine time resolution of TCSPC systems. The parametric model allows the use of continuous-valued parameters, avoiding the construction of a large dictionary to cover many possible combinations of parameter values. Moreover, the closed-form expression is fast to compute, allowing repeated evaluation by our reconstruction algorithm.

While we successfully demonstrate the ERTI acquisition and processing framework here, numerous aspects could be improved through updated experimental and modeling approaches. For instance, because the current system uses a small number of illumination positions, the resulting resolution is coarse. Moreover, the scene modeling constraining objects to extend upward from the ground plane and have constant albedo—although reasonable for a large class of objects, including in outdoor scenes—yields a rough reconstruction, particularly in the vertical dimension, and can lead to errors in reconstructing floating or cantilevered objects, as shown in Supplementary Note 6.

Like most active NLOS methods, ERTI uses a single laser wavelength, thereby not capturing spectral information about the hidden scene. For advanced computer vision techniques that could also benefit from color cues, ERTI could be modified to include multiple laser wavelengths^44^ or a super-continuum laser^45^ and multiple detectors with different spectral filters. Alternatively, because they take advantage of the occlusion effect from identical geometry, ERTI data could be fused with passive corner camera measurements from RGB sensors^22,27,32^.

The acquisition and reconstruction times are not currently fast enough for real-time use. Existing active acquisition systems with higher optical power at the same wavelength^12,15^ could be used to make measurements approximately 10 times faster. Other works have even shown promising results with linear-mode avalanche photodiodes and lasers at longer wavelengths with greater eye-safety^46^. Further algorithm development, especially in faster programming languages or with dedicated processing hardware, would likewise make the presented approach more practical.

Although we assume sequential illumination of evenly-spaced angles and a constant integration time for each spot, an alternative implementation could use a multi-resolution approach that first coarsely captures the hidden scene structure and then more finely samples areas that appear to have interesting content. Finally, ERTI assumes a thin occluding plane with a linear vertical edge. Modified modeling would be required to opportunistically use a wall occluder with non-negligible thickness or other more complicated occluder shapes.

## Methods

### Experimental setup

A 120-mW master oscillator fiber amplifier (MOFA) picosecond laser (PicoQuant VisUV-532) at operating wavelength 532 nm is pulsed with repetition frequency *f*_r_ =  20 MHz. The illumination spot is redirected by a pair of galvo mirrors (Thorlabs GVS012), which is controlled by software through the analog outputs of a data acquisition (DAQ) interface (NI USB-6363). Simultaneously with the illumination trigger, the laser sends a synchronization signal to the TCSPC electronics (PicoQuant HydraHarp 400), which starts a timer. The stop signal for the timer is a detection event registered by the SPAD detector (Micro Photon Devices Fast-gated SPAD, photon detection efficiency  ≈  30% at 532 nm). These detection events may be due to true photon detections such as back-reflected signal or ambient light, or due to noise such as thermal dark counts or afterpulses.

The hardware is positioned approximately 2 m from the occluder edge. The laser illuminates a set of *n*_*ℓ*_ spots ${\{{\ell }_{i}\}}_{i = 1}^{{n}_{\ell }}$ along a semicircle of radius *r*_*ℓ*_ on the floor plane, with the vertical edge at the center. The spots are linearly spaced in angle with *ℓ*_1_ at angle 0 completely occluded from the hidden scene, and ${\ell }_{{n}_{\ell }}$ at angle *π* where none of the hidden scene is occluded.

The SPAD has a 25-mm lens mounted at the focal distance from the detector element, so that the SPAD field of view (FOV) is a small, approximately-circular spot of radius *r*_o_ on the ground plane. The SPAD is mounted on an articulating platform (Thorlabs SL20) and oriented so that the center of the FOV is approximately co-linear with the intersection of the ground plane and the occluding wall, a distance *r*_s_ ≈ 20 cm from the corner. Mounted in front of the collection lens is a bandpass filter (Semrock MaxLine laser-line filter) with a transmission efficiency of  > 90% at the operating wavelength and a full width at half maximum (FWHM) bandwidth of 2 nm to reduce the amount of ambient light incident on the detector. The timing offset of the laser/SPAD system is adjusted such that the round-trip time of flight to and from the corner spot is removed (i.e., the corner point is at time zero). Finally, a gate delay is adjusted so that the first-bounce light from the direct reflection is not recorded, to ensure that afterpulsing due to the strong direct reflection is minimized. The SPAD gating is controlled by a delayer unit (MPD Picosecond Delayer) to have a gate-on duration of 42 ns starting  ≈ 3 ns after the peak of the direct reflection.

The laser is directed by the galvos to illuminate each spot in sequence for a time *t*_dwell_ per spot. Detected photons are time-stamped by TCSPC module and streamed to the computer. When the DAQ changes the galvo voltages to change the coordinates of the laser position, it simultaneously sends a marker to the TCSPC module indicating the spot to which the subsequent detections belong. After the acquisition is completed, a histogram of detection times is formed for time bins with bin centers ${\{{b}_{i}\}}_{i = 1}^{{n}_{{\rm{b}}}}$, where *n*_b_ = ⌊*t*_r_/*t*_bin_⌋ is the number of bins, *t*_bin_ is the bin resolution, and *t*_r_ = 1/*f*_r_ is the repetition period. In this way, histograms can be formed for any histogram dwell time *t*_h_, where *t*_h_ ∈ [0, *t*_dwell_].

## Supplementary information

Supplementary Information

Description of Additional Supplementary Files

Supplementary Movie 1

## Data Availability

Raw data used to produce all experimental figures in the manuscript and supplementary information (Fig. 5 and Supplementary Figs. 10, 11, 13, and 14) is available on GitHub at https://github.com/Computational-Periscopy/ERTI/.

## Code availability

Code used to produce all ERTI reconstruction figures in the manuscript and supplementary information (Figs. 4g and 5 and Supplementary Figs. 12, 13, and 14) is available on GitHub at https://github.com/Computational-Periscopy/ERTI/.
