# Supplementary material for: Seeing around corners with edge-resolved transient imaging
Source: Nat Commun. 2020 Nov 23;11:5929. doi: 10.1038/s41467-020-19727-4 (PMC7683558; doi:10.1038/s41467-020-19727-4)
Supplement: Supplementary file 1 — Supplementary Information [file 41467_2020_19727_MOESM1_ESM.pdf]

Supplementary Information for

Seeing Around Corners with Edge-Resolved Transient Imaging

Joshua Rapp[1,2,†], Charles Saunders[1,†], Julián Tachella[3,†], John Murray-Bruce[1,4], Yoann Altmann[3], Jean-Yves Tourneret[5], Stephen McLaughlin[3], Robin M. A. Dawson[2], Franco N. C. Wong[6], and Vivek K Goyal[1,*]

[1]Department of Electrical and Computer Engineering, Boston University, 1 Silber Way, Boston, Massachusetts 02215, USA
[2]Charles Stark Draper Laboratory, Inc., 555 Technology Square, Cambridge, Massachusetts 02139, USA
[3]School of Engineering and Physical Sciences, Heriot-Watt University, Edinburgh, EH14 4AS, UK
[4]Department of Computer Science and Engineering, University of South Florida, 4202 E. Fowler Avenue, Tampa, Florida 33620, USA
[5]INP/ENSEEHIT-IRIT-TeSA, University of Toulouse, 31071 Toulouse Cedex 7, France
[6]Research Laboratory of Electronics, Massachusetts Institute of Technology, 77 Massachusetts Avenue, Cambridge, Massachusetts 02139, USA
[†]These authors contributed equally.
[*]email: v.goyal@ieee.org

Supplementary Note 1: Transient Light Transport Modeling

The proposed method represents scenes primarily by a collection of vertical facets with four parameters: distance from the vertical edge ρ, height η, albedo α, and orientation ϕ. While such a model is rich enough to describe many outdoor scenes, the model also allows for indoor scenes, which usually have a ceiling, described as a horizontal planar facet parallel to the floor and with height η_c, maximum length ρ_c, and albedo α_c. This note describes how those parameters are mapped to the continuous-time light intensity measured with an idealized pulsed illumination and time-resolved sensing system. Also presented are the mappings for the discrete-time measurement systems used in practice, which integrate the light intensity over bins of duration Δ_t.

We first exploit the inherent symmetry of diffuse light propagation to derive the full response for the intensity from a single wedge based on the response for one half of a fronto-parallel facet ($\phi = 0$). Next, we show how the response for a facet with arbitrary orientation is derived by modifying the distance parameter and linearly combining the responses for two half-facets with different widths based on the orientation angle. If multiple facets are present within a single wedge, closer facets will cause lower parts of the more distant facets to be occluded. We then derive the transient response from the occluded portion of the facet to be subtracted, as well as an approximation to the response from the ceiling. Finally, we how the facet and ceiling components are combined into the full wedge response.

Basic Transient Response Derivation for a Fronto-parallel Half-Facet

Let $\boldsymbol{\ell}$ be the position of a laser illumination, $\mathbf{c}$ be a point in the single-photon avalanche diode (SPAD) field-of-view (FOV), and $\mathbf{p}$ be a point on a hidden surface $\mathcal{S}$. Following previous models for non-line-of-sight (NLOS) imaging [1, 2], the transient light transport due to illumination of $\boldsymbol{\ell}$ at time t_0, which has factors due to the round-trip time of flight, the radial falloff to and from the facet, and cosine factors for

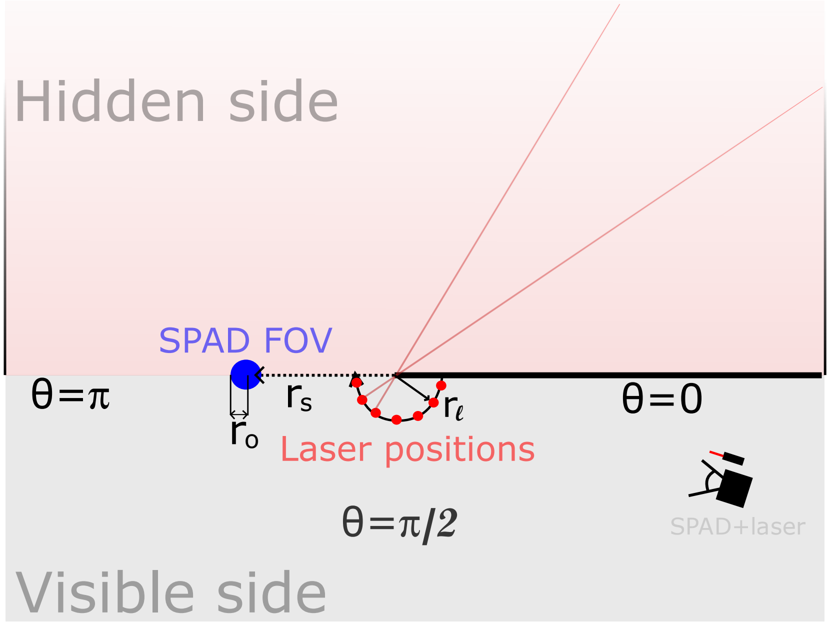

Supplementary Figure 1: Top-down view of the imaging scenario. A laser spot is scanned around a vertical edge along an arc of radius r_ℓ at angles $\theta \in [0, \pi]$. The SPAD detector is focused at at spot with radius r_o a distance r_s from the edge. Time-resolved measurements for each laser position combine light reflected from both visible and hidden sides of the occluding edge. Differences between measurements at each laser position isolate time-of-flight information about individual wedges of the hidden scene.

Lambertian reflection, is given as

$$L(t) = \int_{-\infty}^{t} \int_{\mathcal{S}} \alpha(\mathbf{p}) \frac{G(\mathbf{p}, \boldsymbol{\ell}, \mathbf{c})}{\|\mathbf{p} - \boldsymbol{\ell}\|^2 \|\mathbf{p} - \mathbf{c}\|^2} \delta(t_0 + (\|\mathbf{p} - \boldsymbol{\ell}\| + \|\mathbf{p} - \mathbf{c}\|)/c - t') \, \mathrm{d}\mathbf{p} \, \mathrm{d}t', \tag{1}$$

where $\alpha(\mathbf{p})$ is the albedo at point $\mathbf{p}$, c is the speed of light, the Lambertian bidirectional reflectance distribution function (BRDF) factor is

$$G(\mathbf{p}, \boldsymbol{\ell}, \mathbf{c}) = \cos(\measuredangle(\mathbf{p} - \boldsymbol{\ell}, \mathbf{n}_{\boldsymbol{\ell}})) \cos(\measuredangle(\boldsymbol{\ell} - \mathbf{p}, \mathbf{n}_{\mathbf{p}})) \cos(\measuredangle(\mathbf{c} - \mathbf{p}, \mathbf{n}_{\mathbf{p}})) \cos(\measuredangle(\mathbf{p} - \mathbf{c}, \mathbf{n}_{\mathbf{c}})), \tag{2}$$

$\mathbf{n}_{\boldsymbol{\ell}}$, $\mathbf{n}_{\mathbf{p}}$, and $\mathbf{n}_{\mathbf{c}}$ are the normal vectors of $\boldsymbol{\ell}$, $\mathbf{p}$, and $\mathbf{c}$, respectively, and $\measuredangle(\cdot, \cdot)$ denotes the angle between its vector arguments.

We assume the illumination arc radius r_ℓ, SPAD FOV radius r_o, and the separation between illumination and SPAD FOV spots r_s are small enough such that $\boldsymbol{\ell} \approx \mathbf{c} \approx \mathbf{0}$ (see Supplementary Figure 1). Then the acquisition configuration approximately corresponds to confocal illumination and detection [3] from a single point at the base of the vertical edge, which we define as the origin of our coordinate system. The confocal configuration corresponds to a spherical geometry, such that objects for a given time of flight lie on the same sphere, rather than a more general ellipsoid. We assume that the distance to the corner point is known, so we consider reflection off the floor at time $t_0 = 0$. Supplementary Equation 1 thus simplifies to

$$L(t) = \int_{0}^{t} \int_{\mathcal{S}} \alpha(\mathbf{p}) \frac{G(\mathbf{p}, \mathbf{0}, \mathbf{0})}{\|\mathbf{p}\|^4} \delta(2\|\mathbf{p}\|/c - t') \, \mathrm{d}\mathbf{p} \, \mathrm{d}t'. \tag{3}$$

We consider the light intensity contribution from a single wedge. Our scene representation describes the world as being composed of Lambertian planar facets with a *gravity prior* ensuring all planar facets begin at the ground plane. Without loss of generality, we orient our coordinate system for a wedge so that the facet is centered in the x direction on the y-axis. Our initial modeling is based on a fronto-parallel facet with unit normal vector $\mathbf{n}_{\mathbf{p}} = [0, -1, 0]$. The unit normal vector of the ground is defined as

$\mathbf{n_g} = [0, 0, 1]$. In general, point $\mathbf{p}$ on the facet has coordinates $[x, y, z]$. Thus, we can simplify

$$\begin{aligned} G(\mathbf{p}, \mathbf{0}, \mathbf{0}) &= \cos(\measuredangle(\mathbf{p}, \mathbf{n_g}))\cos(\measuredangle(-\mathbf{p}, \mathbf{n_p}))\cos(\measuredangle(-\mathbf{p}, \mathbf{n_p}))\cos(\measuredangle(\mathbf{p}, \mathbf{n_g})) \\ &= \frac{(\mathbf{p}\cdot\mathbf{n_g})(-\mathbf{p}\cdot\mathbf{n_p})(-\mathbf{p}\cdot\mathbf{n_p})(\mathbf{p}\cdot\mathbf{n_g})}{\|\mathbf{p}\|^4} \\ &= y^2 z^2/\|\mathbf{p}\|^4, \end{aligned} \tag{4}$$

where the dot $(\cdot)$ notation is used here to indicate an inner product.

The geometry for the fronto-parallel facet is shown in Supplementary Figure 2(a). We define the perpendicular distance to the facet as d, so that the closest point to the origin is $[0, d, 0]$, in the center of the bottom facet edge. We define η as the height and α as the uniform albedo of the facet that does not change with position. We consider the transient response of the half-facet in the positive-x half-plane, which has an angular span β. The facet is assumed to span the width of the wedge regardless of the distance, so the half-facet width is $w = d\tan\beta$. For the wedge between θ_{i+1} and θ_i, define the wedge angle as $\Delta_\theta = \theta_{i+1} - \theta_i$, so for the fronto-parallel facet we have $\beta = \Delta_\theta/2$. Finally, define $h(t; d, \eta, \alpha, \beta)$ to be the transient response of the half-facet in the positive-x half-plane. Due to the horizontal symmetry of the facet, we can easily compute the full response of a fronto-parallel facet as

$$L(t) = 2h(t; d, \eta, \alpha, \beta). \tag{5}$$

More importantly, the half-facet response will also be useful for computing responses for arbitrary orientations. To find an expression for the half-facet response, we first expand Supplementary Equation 3 to

$$\begin{aligned} L(t) &= \alpha \int_0^t \int_{\mathcal{S}} \frac{y^2 z^2}{\|\mathbf{p}\|^8}\, \delta(2\|\mathbf{p}\|/c - t')\, \mathrm{d}\mathbf{p}\, \mathrm{d}t' \\ &= \alpha \int_0^t \int_x \int_y \int_z \frac{y^2 z^2}{(x^2+y^2+z^2)^4}\, \delta\!\left(\frac{2}{c}\sqrt{x^2+y^2+z^2} - t'\right) \mathrm{d}z\, \mathrm{d}y\, \mathrm{d}x\, \mathrm{d}t', \end{aligned} \tag{6}$$

so

$$h(t; d, \eta, \alpha, \beta) = \alpha d^2 \int_0^t \int_0^w \int_0^\eta \frac{z^2}{(x^2+d^2+z^2)^4}\, \delta\!\left(\frac{2}{c}\sqrt{x^2+d^2+z^2} - t'\right) \mathrm{d}z\, \mathrm{d}x\, \mathrm{d}t' \tag{7}$$

by introducing the integration limits from the half-facet dimensions (see Supplementary Figure 2(b)).

The spherical propagation of the illumination from the origin intersects a fronto-parallel planar facet along a circle, hence it is convenient to change from Cartesian to cylindrical coordinates (r, ξ, y), where $r^2 = x^2 + z^2$, $z = r\sin\xi$, $x = r\cos\xi$, and $\mathrm{d}z\,\mathrm{d}x = r\,\mathrm{d}r\,\mathrm{d}\xi$. The challenge is in defining the regions of integration. The set of valid radius values is $\mathcal{R} = \{r : 0 \le r \le \max\{w, \eta\}\}$. For a given radial coordinate r, the angles integrated are $\Xi = \left\{\xi : \cos^{-1}(\min\{1, w/r\}) \le \xi \le \sin^{-1}(\min\{1, \eta/r\})\right\}$, giving the transient light transport as

$$h(t; d, \eta, \alpha, \beta) = \alpha d^2 \int_0^t \int_{\mathcal{R}} \int_{\Xi} \frac{r^2\sin^2\xi}{(r^2+d^2)^4}\, \delta\!\left(\frac{2}{c}\sqrt{r^2+d^2} - t'\right) r\, \mathrm{d}\xi\, \mathrm{d}r\, \mathrm{d}t' \tag{8}$$

$$= \alpha d^2 \int_{\mathcal{R}} \frac{r^3}{(r^2+d^2)^4} \int_{\Xi} \sin^2\xi\, \mathrm{d}\xi \int_0^t \delta\!\left(\frac{2}{c}\sqrt{r^2+d^2} - t'\right) \mathrm{d}t'\, \mathrm{d}r \tag{9}$$

$$= \alpha d^2 \int_{\mathcal{R}} \frac{r^3}{(r^2+d^2)^4} \int_{\Xi} \sin^2\xi\, \mathrm{d}\xi\, H\!\left(\frac{2}{c}\sqrt{r^2+d^2} - t\right) \mathrm{d}r, \tag{10}$$

where $H(t)$ is the Heaviside step function.

Practical systems cannot measure the instantaneous transient response. TCSPC systems, for instance, accumulate photon detections within small time intervals (e.g., bins of a histogram). Let $h_t(d, \eta, \alpha, \beta, \Delta_t)$

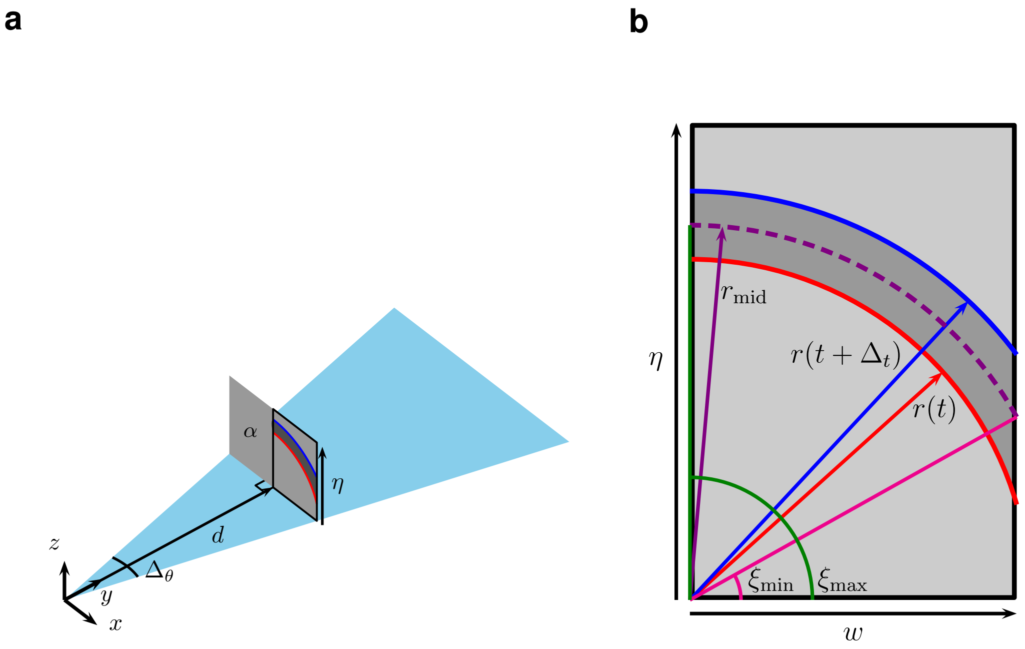

Supplementary Figure 2: The basic transient response within a wedge is computed for one-half of a fronto-parallel planar facet. (a) The fronto-parallel facet is shown at distance d and with height η and albedo α. The half-facet is outlined in bold. (b) A close-up of the half-facet shows the width w computed from the distance d and wedge angle Δ_θ. At time t, the illumination intersects the plane along a circle with radius $r(t)$, and the circle expands to radius $r(t+\Delta_t)$ at time $t+\Delta_t$. We approximate the angular range $[\xi_{\min}, \xi_{\max}]$ based on the averaged circle radius $r_{\mathrm{mid}} = [r(t) + r(t+\Delta_t)]/2$, which causes the integration in Supplementary Equation 12 to become separable and have a closed-form solution.

be the integral of the transient response over a bin of duration Δ_t, i.e.,

$$\begin{aligned} h_t(d, \eta, \alpha, \beta, \Delta_t) &= \int_t^{t+\Delta_t} h(t'; d, \eta, \alpha, \beta)\, \mathrm{d}t' \\ &= \int_t^{t+\Delta_t} \alpha d^2 \int_{\mathcal{R}} \frac{r^3}{(r^2+d^2)^4} \int_{\Xi} \sin^2 \xi \, \mathrm{d}\xi \, H\left(\frac{2}{c}\sqrt{r^2+d^2} - t'\right) \mathrm{d}r\, \mathrm{d}t'. \end{aligned} \quad (11)$$

We can then use the time dependence of the radius $r(t) = \sqrt{(ct/2)^2 - d^2}$. Although the limits of the inner-most integral unfortunately depend on r, the dependence is mild if the time duration Δ_t is small. Thus we fix an approximate angular range to be from $\xi_{\min} = \cos^{-1}(\min\{1, w/r_{\mathrm{mid}}\})$ to $\xi_{\max} = \sin^{-1}(\min\{1, \eta/r_{\mathrm{mid}}\})$, where $r_{\mathrm{mid}} = [r(t) + r(t+\Delta_t)]/2$, as shown in Supplementary Figure 2(b). Thus,

$$h_t(d, \eta, \alpha, \beta, \Delta_t) \approx \alpha d^2 \int_{r(t)}^{r(t+\Delta_t)} \frac{p^3}{(p^2+d^2)^4} \mathbb{1}\left\{\frac{2d}{c} \le t \le \frac{2}{c}\sqrt{d^2+\eta^2+w^2}\right\} \mathrm{d}p \int_{\xi_{\min}}^{\xi_{\max}} \sin^2 \xi \, \mathrm{d}\xi, \quad (12)$$

where $\mathbb{1}$ is the indicator function. The first integral evaluates to

$$\begin{aligned} &\int_{r(t)}^{r(t+\Delta_t)} \frac{p^3}{(p^2+d^2)^4} \mathbb{1}\left\{\frac{2d}{c} \le t \le \frac{2}{c}\sqrt{d^2+\eta^2+w^2}\right\} \mathrm{d}p \\ &\qquad = \frac{1}{12}\left[\frac{3r^2(t)+d^2}{(r^2(t)+d^2)^3} - \frac{3r^2(t+\Delta_t)+d^2}{(r^2(t+\Delta_t)+d^2)^3}\right] \mathbb{1}\left\{\frac{2d}{c} \le t \le \frac{2}{c}\sqrt{d^2+\eta^2+w^2}\right\}, \end{aligned} \quad (13)$$

and the second integral evaluates to

$$\int_{\xi_{\min}}^{\xi_{\max}} \sin^2 \xi \, \mathrm{d}\xi = \frac{1}{2}[\xi_{\max} - \xi_{\min} + \sin(\xi_{\min})\cos(\xi_{\min}) - \sin(\xi_{\max})\cos(\xi_{\max})]. \tag{14}$$

Thus, for each time bin $[t, t+\Delta_t]$, we can compute

$$\begin{aligned} h_t(d, \eta, \alpha, \beta, \Delta_t) \approx \frac{\alpha d^2}{24} & [\xi_{\max} - \xi_{\min} + \sin(\xi_{\min})\cos(\xi_{\min}) - \sin(\xi_{\max})\cos(\xi_{\max})] \\ & \cdot \left[\frac{3r^2(t) + d^2}{(r^2(t)+d^2)^3} - \frac{3r^2(t+\Delta_t)+d^2}{(r^2(t+\Delta_t)+d^2)^3} \right] \mathbb{1}\left\{ \frac{2d}{c} \leq t \leq \frac{2}{c}\sqrt{d^2+\eta^2+w^2} \right\}. \end{aligned} \tag{15}$$

Response Generalization to Arbitrary Orientation

A facet is fronto-parallel when it is perpendicular to the line defining the center of a wedge. In Supplementary Equation 5, we determined the response of a fronto-parallel facet by adding the identical responses of two fronto-parallel half-facets. For a facet that is not fronto-parallel, we can easily determine the response by taking advantage of the same calculations for fronto-parallel half-facets, but combining them differently. Observe that a facet that is not fronto-parallel within a wedge can still be seen as a sum or difference of fronto-parallel facets by rotating the coordinate system to align with the position of the point at the base of an extension of the planar facet where the normal vector points toward the origin. If that point is within the wedge, we have a sum (e.g., addition of half-facets of width w_1 and w_2 in Supplementary Figure 3(a)); if that point is outside the wedge, we have a difference (e.g., half-facet of width w_2 subtracted from a half-facet of width w_1 in Supplementary Figure 3(b)). The closest point on the facet is no longer simply given as the distance to the facet along the y-axis. Instead, we define ρ to be the shortest distance from the origin to the facet. The particular nearest point depends on the magnitude $|\phi|$ relative to the wedge angular extent. Specifically, if $|\phi| < \Delta_\theta/2$ (see Supplementary Figure 3(a)), the closest point to the origin is along the base of the facet, whereas if $|\phi| \geq \Delta_\theta/2$, the closest point to the origin is at one of the bottom corners of the facet (see Supplementary Figure 3(b)). This definition was chosen so that the transient response for facets with the same value of ρ but different orientation angles ϕ would still start at the same time, which helps with the convergence of the MCMC sampler.

The full response for a general facet linearly combines the responses for two fronto-parallel facets with different widths. First, we determine the perpendicular distance to the facet to be $\gamma = \rho\cos(\max\{0, |\phi| - \Delta_\theta/2\})$, which may be different from ρ if $|\phi| > \Delta_\theta/2$. The angular spans of the two half-facets are $\beta_1 = |\Delta_\theta/2 - |\phi||$ and $\beta_2 = \Delta_\theta/2 + |\phi|$, yielding half-facet widths w_1 and w_2 corresponding to $w = \rho\tan\beta$. Finally, the generic facet response is given as

$$g_t(\rho, \eta, \alpha, \phi, \Delta_\theta, \Delta_t) = h_t(\gamma, \eta, \alpha, \beta_2, \Delta_t) + \mathrm{sign}(\Delta_\theta/2 - |\phi|) \cdot h_t(\gamma, \eta, \alpha, \beta_1, \Delta_t). \tag{16}$$

Note that the full response from a fronto-parallel facet is a special case where $\phi = 0$ and matches the expression found in the manuscript.

Incorporation of Occlusion Effect for Multiple Facets Within a Wedge

If multiple facets are present within a single wedge, then closer facets occlude the lower parts of farther facets, so the responses from multiple facets do not combine linearly. To deal with occlusion, we compute the response from each facet in order of the proximity to the origin (i.e., sorted by ρ). We then determine whether or how much of the later facets are visible, given the occlusion effects of closer facets. For instance, Supplementary Figure 4 shows an example in which part of the second facet is still visible. We thus compute the coordinates of the corners of the occluded region, approximate the response of the trapezoidal occluded region as the average of rectangular facet responses, and subtract the response of the occluded region from the total response.

Building on the previous section, we have defined the perpendicular distance to the facet as γ. Unless the facet is fronto-parallel (i.e., $\phi = 0$), one of the facet bottom corners will be closer than the other. The

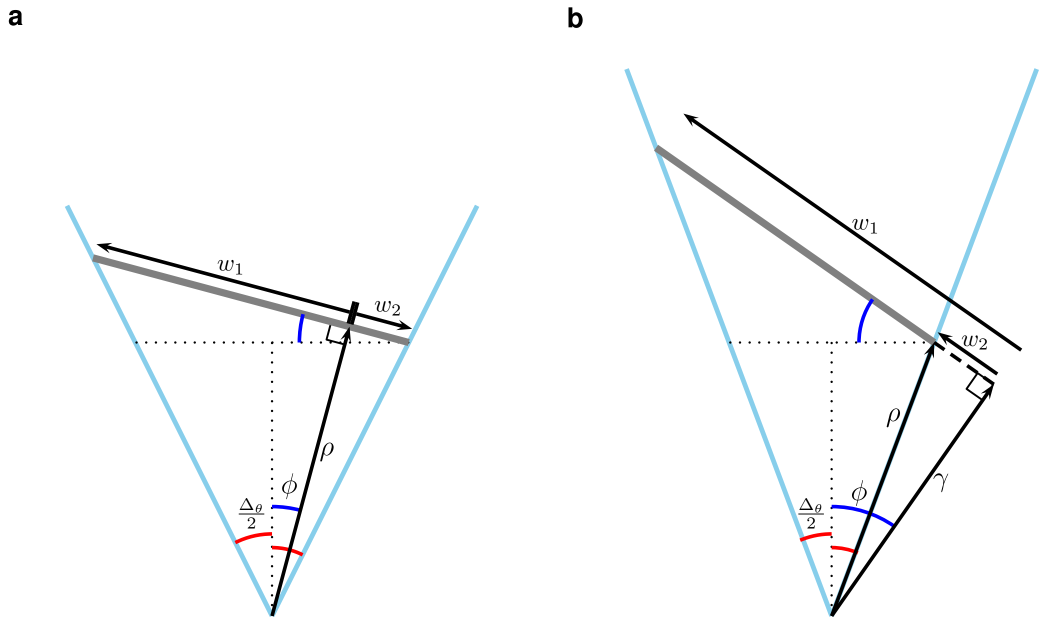

Supplementary Figure 3: Top-down view showing parameter definitions for the facet response with rotation angle ϕ. (a) If $|\phi| < \Delta_\theta/2$, the responses for half-facets of lengths w_1 and w_2 are added. (b) Else if $|\phi| > \Delta_\theta/2$, the response for the half-facet of lengths w_2 is subtracted from the response for the half-facet of lengths w_1.

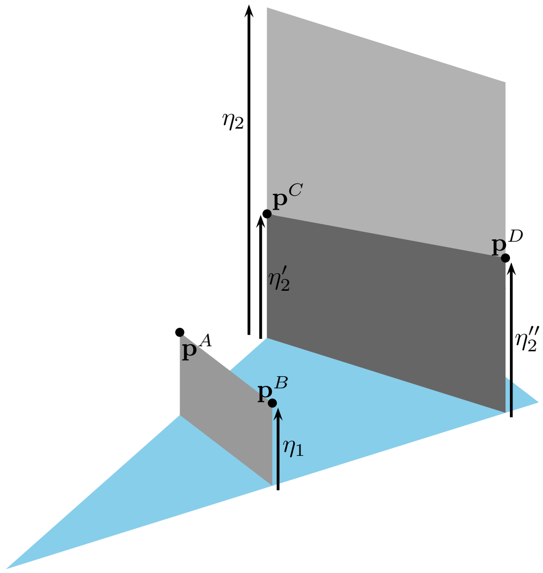

Supplementary Figure 4: The response from a partially-occluded facet is computed by subtracting the approximate response from the occluded region. The occluded region approximation is determined by averaging the responses for facets of height η_2' and η_2''.

closer corner is at a radial distance $r_1 = \gamma/[\cos(||\phi| - \Delta_\theta/2|)]$, and the farther corner is at radial distance $r_2 = \gamma/[\cos(||\phi| + \Delta_\theta/2|)]$. Taking the case that the right corner is closer (i.e., $\phi > 0$), the cylindrical coordinates ($\mathbf{p} = [p_r, p_\theta, p_z]$) of the top corners of the first facet in Supplementary Figure 4 are given as

$$\mathbf{p}^B = [r_1, \theta_\mathrm{R}, \eta_1], \qquad \mathbf{p}^A = [r_2, \theta_\mathrm{L}, \eta_1], \tag{17}$$

where $\theta_\mathrm{L} = \pi/2 + \Delta_\theta/2$ and $\theta_\mathrm{R} = \pi/2 - \Delta_\theta/2$ use the fact that the positive y-axis is assumed to bisect the wedge.

In general, the region of one vertical plane occluded by another vertical plane will be trapezoidal, unless the planes are parallel (e.g., $\phi_1 = \phi_2$). We approximate the response from the occluded region as the average response for planes of heights η_2' and η_2'', which are the heights of the top corners of the trapezoid. If both $\eta_2' < \eta_2$ and $\eta_2'' < \eta_2$, then the response of the occluded plane can be subtracted from the total response for a plane of height η. Else if $\eta_2' > \eta_2$ or $\eta_2'' > \eta_2$, we assume the unoccluded region contributes negligibly to the response within that wedge.

We use the same approach as before to determine the radial coordinates r^C and r^D of the second facet. The heights of $\mathbf{p}^C$ and $\mathbf{p}^D$ can be easily determined as

$$\eta_2' = p_z^C = p_r^C \cdot \frac{p_z^A}{p_r^A}, \qquad \eta_2'' = p_z^D = p_r^D \cdot \frac{p_z^B}{p_r^B}. \tag{18}$$

Finally, the approximate transient light response from a partially-occluded facet is

$$g_t(\rho_2, \eta_2, \alpha_2, \phi_2, \Delta_\theta, \Delta_t) - \frac{1}{2}\left[g_t(\rho_2, \eta_2', \alpha_2, \phi_2, \Delta_\theta, \Delta_t) + g_t(\rho_2, \eta_2'', \alpha_2, \phi_2, \Delta_\theta, \Delta_t)\right]. \tag{19}$$

Ceiling Response Derivation

A model of the world assuming only fronto-parallel planar facets leaves out the contribution from the ceiling, which is a strong exception to the gravity prior. However, computation of the response from a section of illuminated ceiling is not so different to that of other planar facets and is outlined as follows. We assume an entire wedge of the ceiling plane is illuminated as shown in Supplementary Figure 5 and define η_c to be the constant height of the plane. We also define α_c to be the uniform ceiling albedo and choose ρ_c to be the maximum radial extent of the ceiling for the purpose of the derivation.[1] The ceiling response is then given as $c(t; \rho_\mathrm{c}, \eta_\mathrm{c}, \alpha_\mathrm{c}, \Delta_\theta)$.

Once again, assuming a Lambertian ceiling, the temporal intensity response should be given by Supplementary Equation 3. The major difference is in the computation of the BRDF, since the floor and ceiling are assumed to be parallel to each other (i.e., no slanted ceiling). The normal vector of any ceiling point is now $\mathbf{n_p} = [0, 0, -1]$, so the BRDF factor is

$$G(\mathbf{p}, \mathbf{0}, \mathbf{0}) = z^4/\|\mathbf{p}\|^4. \tag{20}$$

We can then once again expand Supplementary Equation 3 for the ceiling response:

$$\begin{aligned} L(t) &= \alpha_\mathrm{c} \int_0^t \int_\mathcal{S} \frac{z^4}{\|\mathbf{p}\|^8}\, \delta(2\|\mathbf{p}\|/c - t')\, \mathrm{d}\mathbf{p}\, \mathrm{d}t' \\ &= \alpha_\mathrm{c} \int_0^t \int_x \int_y \int_z \frac{z^4}{(x^2+y^2+z^2)^4}\, \delta\!\left(\frac{2}{c}\sqrt{x^2+y^2+z^2} - t'\right) \mathrm{d}z\, \mathrm{d}y\, \mathrm{d}x\, \mathrm{d}t', \end{aligned} \tag{21}$$

so

$$c(t; \rho_\mathrm{c}, \eta_\mathrm{c}, \alpha_\mathrm{c}, \Delta_\theta) = \alpha_\mathrm{c}\eta_\mathrm{c}^4 \int_0^t \int_x \int_y \frac{1}{(x^2+y^2+\eta_\mathrm{c}^2)^4}\, \delta\!\left(\frac{2}{c}\sqrt{x^2+y^2+\eta_\mathrm{c}^2} - t'\right) \mathrm{d}y\, \mathrm{d}x\, \mathrm{d}t'. \tag{22}$$

[1] In practice, the ceiling response tends to decay quickly, so the value of ρ_c is set automatically by the maximum time bin in our algorithm's implementation (see Supplementary Note 2). We thus also ignore the possibility of a facet occluding part of the ceiling within a wedge.

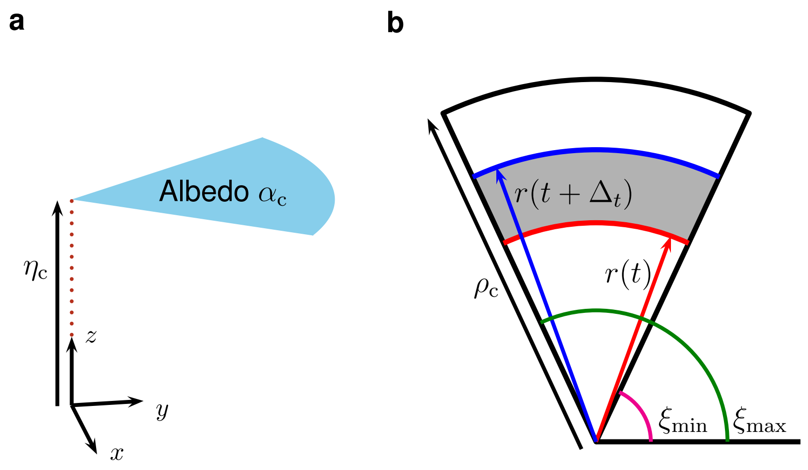

Supplementary Figure 5: Ceiling contribution and forward modeling within a wedge. (a) The entire ceiling plane has height η_c and albedo α_c uniform across all wedges. (b) The maximum radial extent of the ceiling is ρ_c. Integrating the ceiling response yields a closed-form expression.

We once again change to cylindrical coordinates with the longitudinal axis now aligned with the z-axis, so $r^2 = x^2 + y^2$, $x = r\cos\theta$, $y = r\sin\theta$, and $\mathrm{d}x\,\mathrm{d}y = r\,\mathrm{d}r\,\mathrm{d}\theta$. Assuming a maximum distance of the wedge ρ_c, we thus rewrite

$$c(t;\rho_c,\eta_c,\alpha_c,\Delta_\theta) = \alpha_c\eta_c^4 \int_0^t \int_0^{\Delta_\theta} \int_0^{\rho_c} \frac{r}{(r^2+\eta_c^2)^4}\,\delta\!\left(\frac{2}{c}\sqrt{r^2+\eta_c^2} - t'\right) \mathrm{d}r\,\mathrm{d}\theta\,\mathrm{d}t'. \tag{23}$$

Integrating over a time window $[t, t+\Delta_t)$ then produces

$$c_t(\rho_c,\eta_c,\alpha_c,\Delta_\theta,\Delta_t) = \alpha_c\eta_c^4\Delta_\theta \int_{r(t)}^{r(t+\Delta_t)} \frac{r}{(r^2+\eta_c^2)^4}\mathbb{1}\left\{\frac{2\eta_c}{c} \le t \le \frac{2}{c}\sqrt{\eta_c^2+\rho_c^2}\right\}\mathrm{d}r. \tag{24}$$

The remaining integral has a closed-form expression,

$$\int \frac{r}{(r^2+z^2)^4}\,\mathrm{d}r = \frac{-1}{6(r^2+z^2)^3},$$

so the measurement simplifies to

$$c_t(\rho_c,\eta_c,\alpha_c,\Delta_\theta,\Delta_t) = \frac{\alpha_c\eta_c^4\Delta_\theta}{6}\left[\frac{1}{(r^2(t)+z^2)^3} - \frac{1}{(r^2(t+\Delta_t)+\eta_c^2)^3}\right]\mathbb{1}\left\{\frac{2\eta_c}{c} \le t \le \frac{2}{c}\sqrt{\eta_c^2+\rho_c^2}\right\}. \tag{25}$$

Full Wedge Transient Response Computation

The responses from the ceiling and N_i facets in wedge i are combined to compute the full wedge response $f_t(\rho_c,\eta_c,\alpha_c,\boldsymbol{\rho},\boldsymbol{\eta},\boldsymbol{\alpha},\boldsymbol{\phi},\Delta_\theta,\Delta_t)$, where $\boldsymbol{\rho}$, $\boldsymbol{\eta}$, $\boldsymbol{\alpha}$, and $\boldsymbol{\phi}$ are length-N_i vectors. If $N_i \ge 2$, the full wedge response computation includes the non-linear combination due to occlusion:

$$\begin{aligned} &f_t(\rho_c,\eta_c,\alpha_c,\boldsymbol{\rho},\boldsymbol{\eta},\boldsymbol{\alpha},\boldsymbol{\phi},\Delta_\theta,\Delta_t) \\ &\quad = c_t(\rho_c,\eta_c,\alpha_c,\Delta_\theta,\Delta_t) + g_t(\rho_1,\eta_1,\alpha_1,\phi_1,\Delta_\theta,\Delta_t) \\ &\qquad + \sum_{k=2}^{N_i}\left\{g_t(\rho_k,\eta_k,\alpha_k,\phi_k,\Delta_\theta,\Delta_t) - \frac{1}{2}\left[g_t(\rho_k,\eta_k',\alpha_k,\phi_k,\Delta_\theta,\Delta_t) + g_t(\rho_k,\eta_k'',\alpha_k,\phi_k,\Delta_\theta,\Delta_t)\right]\right\}, \end{aligned} \tag{26}$$

which removes the occluded portion of a facet based on the height of the previous facet. It is assumed that the parameter vectors are ordered corresponding to $\rho_1 < \cdots < \rho_k < \cdots < \rho_{N_i}$.

Supplementary Note 2: Reconstruction Algorithm

The reconstruction algorithm is a Bayesian method relying on reversible-jump Markov chain Monte Carlo (RJ-MCMC) simulation. It requires (i) likelihoods for measurements given the set of scene parameters; (ii) prior probability distributions for the scene parameters; and (iii) a set of "moves" for generating samples in steps of the algorithm in order to explore the resulting posterior distribution of the set of scene parameters. Scene parameterization is discussed first. The likelihoods are then derived from the response modeling in Supplementary Note 1. As described next, the prior model is based on natural relationships across wedges, such as continuity of surfaces. Finally, the method for generating samples is described and the full resulting algorithms are presented.

Parameterization

The hidden room is characterized by a ceiling and a set of planar, vertical facets. The ceiling contribution is defined by two parameters: height η_c and albedo α_c, collectively referred to as $\boldsymbol{\chi} = [\eta_c, \alpha_c]^T$. The planar facet indexed by n is described by the 4-tuple $(\mathbf{x}_n, \alpha_n, \eta_n, \phi_n)$, where $\mathbf{x}_n = [\theta_n, \rho_n]^T$ is the position discretized by the n_ℓ illumination spots and n_b time bins[2], $\alpha_n \in \mathbb{R}_+$ is the (unnormalized) albedo, $\eta_n \in \mathbb{R}_+$ is the height, and $\phi_n \in [0, \pi/2]$ the orientation angle. The set of all planar facets across all wedges is defined as

$$\Phi = \{(\mathbf{x}_n, \alpha_n, \eta_n, \phi_n), \quad n = 1, \ldots, N_\Phi\}. \tag{27}$$

Here we interpret the set of facets Φ as a realization of a point process defined in 2D space $[1, n_\ell] \times [1, n_b]$, where each point has additional *marks* (i.e., properties) that are the height, albedo, and orientation of the facet. Using a marked point process model, we do not fix a number N_Φ a priori. To simplify notation, we denote the set of point coordinates (without marks) as $\Phi_\mathbf{x} = \{\mathbf{x}_n, n = 1, \ldots, N_\Phi\}$.

Likelihood

Assuming the incoming light flux incident on the single-photon detector is sufficiently low, the measured photon counts $m_{i,t}$ at bin t for the ith illumination spot follow a Poisson distribution,

$$m_{i,t} \,|\, \Phi, \boldsymbol{\chi}, v_t, b \sim \mathcal{P}\left(\sum_{j=1}^{i} u_{j,t} + v_t + b\right), \tag{28}$$

where $u_{j,t} = u_{j,t}(\Phi, \boldsymbol{\chi})$ is the hidden scene component in the jth wedge and histogram bin t that is approximately described by the facet modeling and the ceiling parameters via Supplementary Equation 26, v_t is the contribution from the visible side at bin t (assumed to be the same across illumination spots), and b is the background level due to ambient illumination. The histogram differences $y_{i,t} = m_{i+1,t} - m_{i,t}$ follow a Skellam[3] distribution [4],

$$y_{i,t} \,|\, \Phi, \boldsymbol{\chi} \sim \text{Skellam}(u_{i,t}, \sigma_{i,t}^2), \tag{29}$$

with mean $u_{i,t}$ and variance

$$\sigma_{i,t}^2 = u_{i,t} + 2\left(\sum_{j=1}^{i-1} u_{j,t} + v_t + b\right). \tag{30}$$

To improve the convergence properties of the MCMC algorithm and reduce its computational cost, we have observed that it is preferable to work with the differences $y_{i,t}$ instead of the original measurements $m_{i,t}$. This observation will be confirmed in Section 3 by simulation. Although the mean of the Skellam

[2] The facet position in meters is obtained using the time bin duration Δ_t and wedge angle of the system Δ_θ.

[3] The Skellam probablity mass function is defined as $p(x; \mu, \sigma) = \exp(-\sigma^2)\left(\frac{\sigma^2+\mu}{\sigma^2-\mu}\right)^{x/2} I_x(\sqrt{\sigma^4 - \mu^2})$, where I_x the modified Bessel function of the first kind.

distribution in Supplementary Equation 29 is only $u_{i,t}$ (i.e., only the wedge i), the variance $\sigma^2_{i,t}$ depends on the configuration of the scene in the previous wedges. This prevents the model in Supplementary Equation 29 from being separable. Here, we first approximate the distribution of the measurements $y_{i,t}$ using composite marginal likelihoods [5]. More precisely, the joint likelihood of histogram differences $\mathbf{Y} \in \mathbb{Z}^{n_b \times n_\ell}$, with $[\mathbf{Y}]_{i,t} = y_{i,t}$, is approximated using separable factors

$$p(\mathbf{Y} \,|\, \Phi, \boldsymbol{\chi}) \approx \prod_{i=1}^{n_b} \prod_{t=1}^{n_\ell} P(y_{i,t} \,|\, \Phi, \boldsymbol{\chi}), \tag{31}$$

where $P(y_{i,t} \,|\, \Phi, \boldsymbol{\chi})$ is the Skellam probability mass given by Supplementary Equation 29 and the variance is approximated by $\sigma^2_{i,t} \approx m_{i+1,t} + m_{i,t}$. Using this first approximation, a hidden facet only affects the measurements linked to its wedge, instead of the complete data, allowing local likelihood evaluation and parameter updates. Moreover, the estimation of the visible and background contributions can be bypassed. Without loss of reconstruction performance, we further approximate the Skellam factors using their first two moments (i.e., a Gaussian density). This second approximation further reduces the computational cost associated with the likelihood evaluation. An experimental comparison between the full likelihood and its separable approximations is presented in Section 3, validating the efficiency of these approximations. Interestingly, we have empirically observed that the separable likelihood model in Supplementary Equation 31 is more robust to imperfections in the sensing process (e.g., non-ideal occlusion) and mismatch between the recorded wedge response and the transient response model computed with Supplementary Equation 26.

Prior Distributions

Facet Positions

We use a spatial point process prior distribution for the positions of the facets, similar to the one developed in the ManiPoP algorithm [6]. While ManiPoP defines a prior for 2D manifolds embedded in a 3D space, here we use similar ideas to model 1D manifolds in 2D space (wedge and depth coordinates). This prior model is designed to promote spatial correlation between facets within the same object (e.g., a wall) and repulsion between facets in the same wedge belonging to different objects (e.g., mannequin and wall in the same wedge). Supplementary Figure 6 illustrates these phenomena. The spatial point process prior is defined as a density f with respect to a Poisson point process reference measure [7, Chapter 9],

$$f(\Phi_{\mathbf{x}} \,|\, d_{\min}, \gamma_a, \lambda_a) \propto f_1(\Phi_{\mathbf{x}} \,|\, d_{\min}) f_2(\Phi_{\mathbf{x}} \,|\, \gamma_a, \lambda_a), \tag{32}$$

where $f_1(\Phi_{\mathbf{x}} \,|\, d_{\min})$ and $f_2(\Phi_{\mathbf{x}} \,|\, \gamma_a, \lambda_a)$ are the Strauss and area interaction [8] processes, respectively. The repulsive Strauss process is defined as

$$f_1(\Phi_{\mathbf{x}} \,|\, d_{\min}) \propto \begin{cases} 0, & \text{if there exists } n \neq n' \text{ such that } \theta_n = \theta_{n'} \text{ and } |\rho_n - \rho_{n'}| < d_{\min}; \\ 1, & \text{otherwise,} \end{cases} \tag{33}$$

where $d_{\min}$ is the minimum separation in histogram bins between two facets in the same wedges. Attraction between points within the same surface is promoted by the area interaction process,

$$f_2(\Phi_{\mathbf{x}} \,|\, \gamma_a, \lambda_a) \propto \lambda_a^{N_\Phi} \gamma_a^{-m\left(\bigcup_{n=1}^{N_\Phi} S(\mathbf{x}_n)\right)}, \tag{34}$$

where $m(\cdot)$ denotes the standard counting measure, $S(\mathbf{x}_n)$ defines a set around the point $\mathbf{x}_n$, and γ_a and λ_a are two hyperparameters, accounting for the amount of attraction and total number of facets, respectively. Both densities define Markovian interactions between points, only correlating points in a local neighbourhood (left and right wedges). Moreover, the combination of both processes implicitly defines a connected-facet structure, which is used to model 1D manifolds in a (discrete) 2D space.

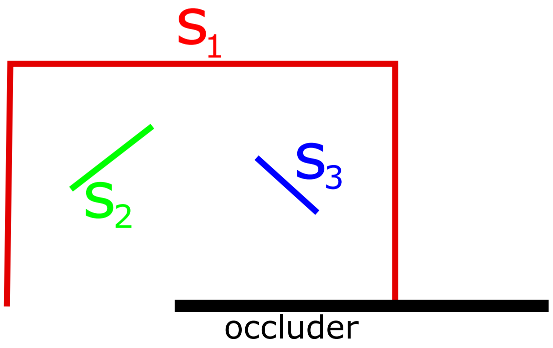

Supplementary Figure 6: Top-down view of a hidden scene's 1D manifold structure. The facets of the room can be clustered together into 3 different 1D curves, i.e. S_1, S_2 and S_3. The position of the facets within each curve is highly correlated with its neighbours. We model these correlations with an area interaction process. Moreover, the different curves are usually clearly separated (in depth) within each wedge. This property is modelled with a repulsive Strauss process.

Facet Parameters

Since facets in neighbouring wedges usually correspond to the same hidden object (e.g., the walls, a piece of furniture, etc.), their albedo, height and angle are generally correlated. We model these correlations with Gaussian Markov random fields, making use of the implicit connected structure, which only promotes correlation between facets linked to the same object. To accommodate the compact support of the marks, we introduce a change of variables that maps a compactly supported random variable into one defined on $\mathbb{R}$, which is a standard procedure in spatial statistics [9]. In our case, we define the mappings

$$\tilde{\alpha}_n = \log \alpha_n - \mu_\alpha, \tag{35}$$

$$\tilde{\eta}_n = \log \eta_n - \mu_\eta, \tag{36}$$

$$\tilde{\phi}_n = \log\left(\frac{\phi_n}{\frac{\pi}{2} - \phi_n}\right) - \mu_\phi, \tag{37}$$

where μ_α, μ_η, and μ_ϕ are constants that center the distributions around the origin. The prior distribution of the log-albedo is then defined by

$$\tilde{\alpha} \,|\, \sigma^2_{\tilde{\alpha}}, \beta_{\tilde{\alpha}}, \rho, \Phi_{\mathbf{x}} \sim \mathcal{N}(\mathbf{0}, \sigma^2_{\tilde{\alpha}} \mathbf{P}^{-1}), \tag{38}$$

where $\tilde{\alpha} = [\tilde{\alpha}_1, \dots, \tilde{\alpha}_{N_\Phi}]^T$ is a vector containing all the intensities, and $\sigma^2_{\tilde{\alpha}}$, $\beta_{\tilde{\alpha}}$, and ρ are hyperparameters controlling the level of smoothness. The precision matrix $\mathbf{P}$ is defined by

$$[\mathbf{P}]_{n,n'} = \begin{cases} \beta + \sum_{\tilde{n} \in \mathcal{M}_{pp}(\mathbf{x}_n)} \rho, & \text{if } n = n'; \\ -\rho, & \text{if } \mathbf{x}_n \in \mathcal{M}_{pp}(\mathbf{c}_{n'}); \\ 0, & \text{otherwise,} \end{cases} \tag{39}$$

where $\mathcal{M}_{pp}(\mathbf{c}_{n'})$ is the set of neighbours of point $\mathbf{x}_n$, which is obtained using the connected-surface structure. Note that the matrix $\mathbf{P}$ is very sparse due to the Markovian structure of the prior model. Similar prior distributions are assigned to the transformed heights $\tilde{\eta}$ and angles $\tilde{\phi}$, and they are defined by the hyperparameters $(\sigma^2_{\tilde{\eta}}, \beta_{\tilde{\eta}})$ and $(\sigma^2_{\tilde{\phi}}, \beta_{\tilde{\phi}})$, respectively.

Ceiling Parameters

We assign gamma prior distributions to the height and albedo of the ceiling,

$$\eta_c \,|\, k_\eta, \theta_\eta \sim \mathcal{G}(k_\eta, \theta_\eta), \tag{40}$$

$$\alpha_c \,|\, k_\alpha, \theta_\alpha \sim \mathcal{G}(k_\alpha, \theta_\alpha), \tag{41}$$

where (k_η, θ_η) and $(k_\alpha, \theta_\alpha)$ are hyperparameters. This choice is mainly motivated by the fact that it ensures the positivity of the height and albedo.

Parameter Estimation

Using the measurement likelihood from Supplementary Equation 31 and the priors from Supplementary Equations 32 through 41, the posterior distribution of the hidden room parameters (planar facets and ceiling) given the observations $\mathbf{Y}$ is

$$p(\Phi, \boldsymbol{\chi} \,|\, \mathbf{Y}, \boldsymbol{\Psi}) \;\propto\; p(\mathbf{Y} \,|\, \Phi, \boldsymbol{\chi})\, p(\tilde{\alpha} \,|\, \Phi_{\mathbf{x}}, \sigma^2_{\tilde{\alpha}}, \beta_{\tilde{\alpha}})\, p(\tilde{\eta} \,|\, \Phi_{\mathbf{x}}, \sigma^2_{\tilde{\eta}}, \beta_{\tilde{\eta}}) \cdots$$
$$p(\tilde{\phi} \,|\, \Phi_{\mathbf{x}}, \sigma^2_{\tilde{\phi}}, \beta_{\tilde{\phi}})\, f(\Phi_{\mathbf{x}} \,|\, d_{\min}, \gamma_a, \lambda_a)\, \pi(\Phi_{\mathbf{x}})\, p(\eta_{\mathrm{c}} \,|\, k_\eta, \theta_\eta)\, p(\alpha_{\mathrm{c}} \,|\, k_\alpha, \theta_\alpha), \quad (42)$$

where $\boldsymbol{\Psi} = \{\sigma^2_{\tilde{\alpha}}, \beta_{\tilde{\alpha}}, \sigma^2_{\tilde{\eta}}, \beta_{\tilde{\eta}}, \sigma^2_{\tilde{\phi}}, \beta_{\tilde{\phi}}, k_\eta, \theta_\eta, k_\alpha, \theta_\alpha, d_{\min}, \gamma_a, \lambda_a\}$ defines the set of (fixed) hyperparameters. The hyperparameters have been set to

$$\boldsymbol{\Psi} = \{2.25,\ 11.25,\ 2.25,\ 11.25,\ 1,\ 5,\ 10,\ 0.5,\ 2,\ 1,\ 0.17c\Delta_t,\ e^4,\ e^{n_\ell/4}\},$$

reflecting our prior knowledge about the unknown parameters (e.g., the ceiling height should be approximately between 2 and 8 metres, the minimum distance between 2 facets within a wedge is 35 centimetres, etc.).

Rather than the full posterior distribution in Supplementary Equation 42, in this work, we compute the same posterior statistics as in [6]: the facet parameters are estimated using the maximum a posteriori (MAP) estimator

$$\arg\max_{\Phi, \boldsymbol{\chi}} p(\Phi, \boldsymbol{\chi} \,|\, \mathbf{Y}, \boldsymbol{\Psi}), \quad (43)$$

and the ceiling parameters are estimated using the minimum mean squared error estimator

$$\mathbb{E}\{\boldsymbol{\chi} \,|\, \mathbf{Y}, \boldsymbol{\Psi}\}. \quad (44)$$

As these estimators cannot be derived analytically, we resort to Markov chain Monte Carlo (MCMC) simulation methods. The simulation method, discussed below, generates N_m samples of $(\Phi, \boldsymbol{\chi})$,

$$\{\Phi^{(s)}, \boldsymbol{\chi}^{(s)}, \quad \text{for } s = 0, 1, \ldots, N_m - 1\}, \quad (45)$$

that are approximately drawn from the posterior distribution in Supplementary Equation 42. These samples are then used to approximate the estimators, The estimate of the facet set is given by $\widehat{\Phi} = \Phi^{(\hat{s})}$, where

$$\hat{s} = \arg\max_{s \in \{0, \ldots, N_m - 1\}} p(\Phi^{(s)}, \boldsymbol{\chi}^{(s)} \,|\, \mathbf{Y}, \boldsymbol{\Psi}).$$

The estimate of the ceiling is given by

$$\widehat{\boldsymbol{\chi}} = \frac{1}{N_m - N_{\mathrm{bi}}} \sum_{s = N_{\mathrm{bi}}+1}^{N_m} \boldsymbol{\chi}^{(s)},$$

where N_{bi} is the number of burn-in iterations.

Reversible-Jump Markov Chain Monte Carlo Sampling

As in ManiPoP [6], we use a reversible-jump MCMC algorithm that can handle the varying-dimension nature of the spatial point process and allows us to build proposals tailored for the reconstruction problem; see [7] for other stochastic simulation algorithms that can handle varying dimensions. RJ-MCMC can be interpreted as a natural extension of the Metropolis-Hastings algorithm for problems with an a priori unknown dimensionality. Due to the separable Skellam model and Markovian nature of the prior distributions, all the proposed moves are local, having complexities proportional to the size of a single histogram of differences. These moves are detailed in the following paragraphs. For ease of presentation, we summarize the main aspects of each move, referring the reader to [6] for a more detailed description of the different moves.

p_{birth}	1/24	p_{death}	1/24	p_{dilation}	4/24
p_{erosion}	4/24	p_{shift}	1/24	p_{mark}	13/24

Supplementary Table 1: Move probabilities used in the RJ-MCMC sampler.

Birth and death moves. The birth move proposes a new point, sampled uniformly at random in the 2D space. The albedo, height, and orientation angle are sampled from their prior distributions in Supplementary Equation 38. The complementary move, death, proposes the removal of a facet chosen uniformly at random.

Dilation and erosion moves. Birth moves can suffer from low acceptance ratio, as the probability of randomly proposing a facet in the correct position can be low. However, this problem can be overcome by using the current estimation of the surface to propose new facets in regions of high probability. The dilation move proposes a point inside the neighbourhood of an existing surface with uniform probability across all possible neighbouring positions where a point can be added. The rest of the parameters of the new facet (albedo, height, and orientation angle) are proposed in the same way as in the birth move (sampled from their prior distributions). The complementary move (named erosion) proposes to remove a point with one or more neighbours.

Mark and shift moves. As in ManiPoP, the mark move updates the log-albedo of a randomly chosen point $\mathbf{x}_n$. The albedo of the facet is updated independently using a Gaussian proposal with variance $\delta_{\tilde{\alpha}}$,

$$\tilde{\alpha}_n' \sim \mathcal{N}\left(\tilde{\alpha}_n, \delta_{\tilde{\alpha}}\right). \tag{46}$$

Similarly, the shift move updates the position of a uniformly chosen point within a wedge using a Gaussian proposal with variance δ_ρ,

$$\rho_n' \sim \mathcal{N}\left(\rho_n, \delta_\rho\right). \tag{47}$$

The values of $\delta_{\tilde{\alpha}}$ and δ_ρ are adjusted by cross-validation to yield an acceptance ratio close to 41% for each move, which is the optimal value for a one dimensional Metropolis random walk, as explained in [7, Chapter 4].

Sampling the ceiling parameters. The ceiling parameters are also sampled using a Metropolis random walk step, as in the mark and shift moves, i.e.,

$$\boldsymbol{\chi}' \sim \mathcal{N}\left(\boldsymbol{\chi}, \mathrm{diag}(\delta_{\eta_c}, \delta_{\alpha_c})\right). \tag{48}$$

Full SkellaPoP Algorithms

The RJ-MCMC algorithm chooses a different move at each iteration according to the probabilities in Supplementary Table 1 and updates the ceiling parameters every $n_\ell n_b$ (total number of histogram bins) iterations. The resulting algorithm, whose pseudocode is presented in Algorithm 1, is referred to as SkellaPoP, as it handles measurements corrupted by Skellam noise and models the unknown parameters with a point process.

To speed up the convergence of the RJ-MCMC algorithm, we adopt a multiresolution approach in a fashion similar to [6]. The dataset is downsampled by integrating photon detections in groups of 2 wedges. Hence, the number of wedges is reduced by a factor of 2, leading to fewer points and background levels to infer with 2 times more photons per wedge. The estimated point cloud at the coarse scale is upsampled using a simple nearest neighbour algorithm and used as initialization for the next (finer) scale. In all our experiments we repeat the process for $K = 2$ scales. The multiresolution approach is finally summarized in Algorithm 2. The ceiling parameters are initialized by first integrating the histogram differences across all wedges, as all the ceiling contributions are approximately equal across wedges. The first estimate of the ceiling's height is obtained by finding the maximum likelihood estimate of the integrated data, in a similar fashion to matched filtering.

Algorithm 1 SkellaPoP

Input: Histogram differences $\mathbf{Y}$, initial estimate $(\Phi^{(0)}, \boldsymbol{\chi}^{(0)})$ and hyperparameters $\boldsymbol{\Psi}$
Initialization:
$(\Phi, \boldsymbol{\chi}) \leftarrow (\Phi^{(0)}, \boldsymbol{\chi}^{(0)})$
$s \leftarrow 0$
$\widehat{\boldsymbol{\chi}} \leftarrow 0$
Main loop:
while $s < N_m$ **do**
  if $\mathrm{rem}(s, n_\ell n_b) == 0$ **then**
    $(\boldsymbol{\chi}, \delta_{\mathrm{map}}) \leftarrow$ sample $\boldsymbol{\chi}$ using Supplementary Equation 48
  end if
  Choose move according to probabilities in Supplementary Table 1
  $(\Phi, \delta_{\mathrm{map}}) \leftarrow$ perform selected move
  $\mathrm{map} \leftarrow \mathrm{map} + \delta_{\mathrm{map}}$
  if $s \geq N_{\mathrm{bi}}$ **then**
    $\widehat{\boldsymbol{\chi}} \leftarrow \widehat{\boldsymbol{\chi}} + \boldsymbol{\chi}$
    if $\mathrm{map} > \mathrm{map}_{\max}$ **then**
      $\widehat{\Phi} \leftarrow \Phi$
      $\mathrm{map}_{\max} \leftarrow \mathrm{map}$
    end if
  end if
  $s \leftarrow s + 1$
end while
$\widehat{\boldsymbol{\chi}} \leftarrow \widehat{\boldsymbol{\chi}} / (N_m - N_{\mathrm{bi}})$
Output: Final estimates $(\widehat{\Phi}, \widehat{\boldsymbol{\chi}})$

Algorithm 2 Multiresolution SkellaPoP

Input: Data $\mathbf{Y}$, hyperparameters $\boldsymbol{\Psi}$ and number of scales K.
Initialization:
$\Phi_1^{(0)} \leftarrow \emptyset$ (empty room)
$\boldsymbol{\chi}_1^{(0)} \leftarrow$ initialisation with integrated differences
Main loop:
for $k = 1, \ldots, K$ **do**
  if $k > 1$ **then**
    $(\Phi_k^{(0)}, \boldsymbol{\chi}_k^{(0)}) \leftarrow \mathrm{upsample}(\widehat{\Phi}_{k-1}, \widehat{\boldsymbol{\chi}}_{k-1})$
  end if
  $(\widehat{\Phi}_k, \widehat{\boldsymbol{\chi}}_k) \leftarrow \mathrm{SkellaPoP}(\mathbf{Y}_k, \Phi_k^{(0)}, \boldsymbol{\chi}_k^{(0)}, \Psi)$
end for
Output: $(\widehat{\Phi}_K, \widehat{\boldsymbol{\chi}}_K)$

Supplementary Note 3: Observation Model Comparison

We have compared the performance of the RJ-MCMC sampler using the original likelihood in Supplementary Equation 28 and the composite likelihood in Supplementary Equation 31, considering the following options:

1. All scales Poisson: Correlated Poisson model in Supplementary Equation 28 at all scales. Note that this model is strictly equivalent to the Skellam-based model using difference histograms as input data and correlated variances.

2. Skellam: Separable composite likelihood with Skellam factors of variance $\sigma^2_{i,t} \approx m_{i+1,t} + m_{i,t}$ at all scales.

3. Gaussian: Separable composite likelihood with Gaussian factors of variance $\sigma^2_{i,t} \approx m_{i+1,t} + m_{i,t}$ at all scales.

4. Skellam denoised variance: Separable composite likelihood with Skellam terms of variance obtained by denoising $m_{i+1,t} + m_{i,t}$ under a temporal smoothness assumption.

5. Gaussian denoised variance: Separable composite likelihood with Gaussian terms of variance obtained by denoising $m_{i+1,t} + m_{i,t}$ under a temporal smoothness assumption.

6. Fine-scale Poisson: We run the sampler using the separable composite likelihood in the coarse scale and the correlated Poisson model in the fine scale. The coarse-scale estimate provides a good initialization for the fine scale, improving the mixing of the RJ-MCMC algorithm based on the correlated likelihood.

We simulated a synthetic room using the full model in Supplementary Equation 28 to evaluate these six alternatives using the same number of scales and Monte Carlo iterations. The room is composed of three walls, a ceiling, and one central object, which partially occludes one of the walls. Supplementary Figure 7 shows the performance of the evaluated algorithms, as a function of the mean number of recorded photons per histogram bin.

The algorithms based on the full non-separable likelihood find more false detections and fail to find all the facets, even at high photon counts. This behaviour can be attributed to the bad mixing of the resulting sampler, which is more easily stuck in a local maximum. Moreover, the execution time of these algorithms is significantly larger than that of the algorithms using composite likelihoods, as each likelihood evaluation (in the RJ-MCMC moves) requires a computation over the complete dataset of order $\mathcal{O}(n_\ell n_b)$. In contrast, the separable models only require a computation of order $\mathcal{O}(n_b)$. Both for the Skellam and Gaussian separable models, no significant improvement can be attributed to pre-estimating the variance with a denoiser. The Gaussian approximation performs better than the Skellam model, possibly due to the numerical instability of the numerical evaluation of the Skellam probability mass function. Moreover, the computation of the Skellam likelihood requires more operations, resulting in longer execution times than the Gaussian alternative. Hence, we have used the separable Gaussian model in the rest of our experiments.

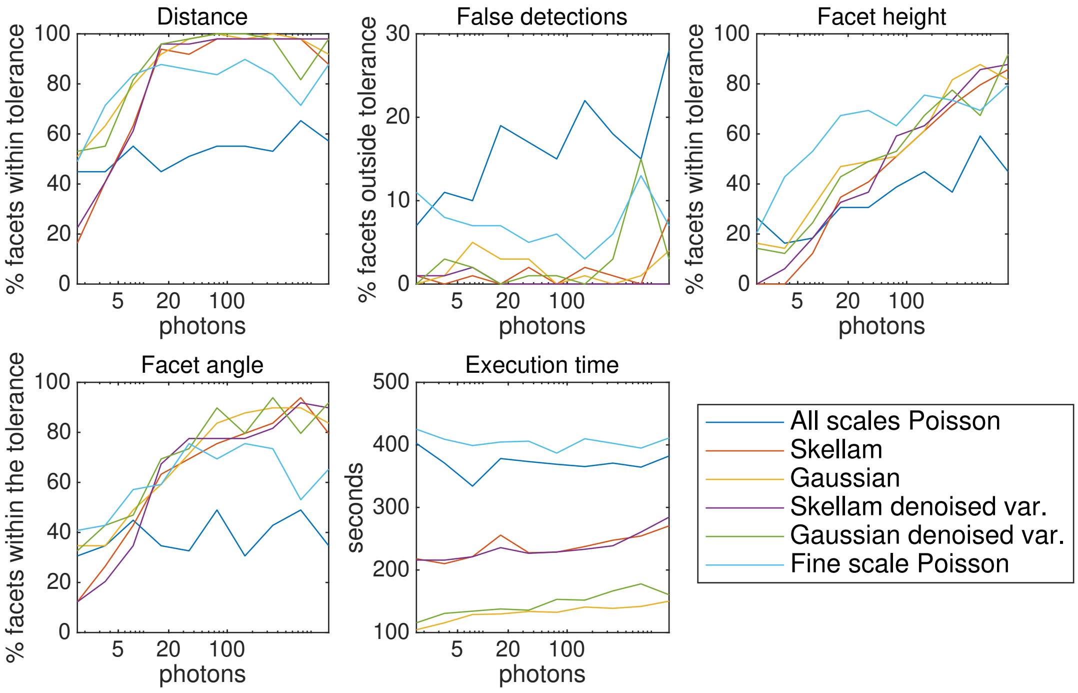

Supplementary Figure 7: Impact of the observation model on the reconstruction performance. The results are shown for a tolerance of 20 cm for the distance, 50 cm for the facet height and 30 degrees for the orientation angle.

Supplementary Note 4: Additional Experimental Details

Please see the Methods for a complete description of the experimental equipment. The experimental setup used for data collection is shown in Supplementary Figure 8. The FOV from the equipment location is shown in Supplementary Figure 9.

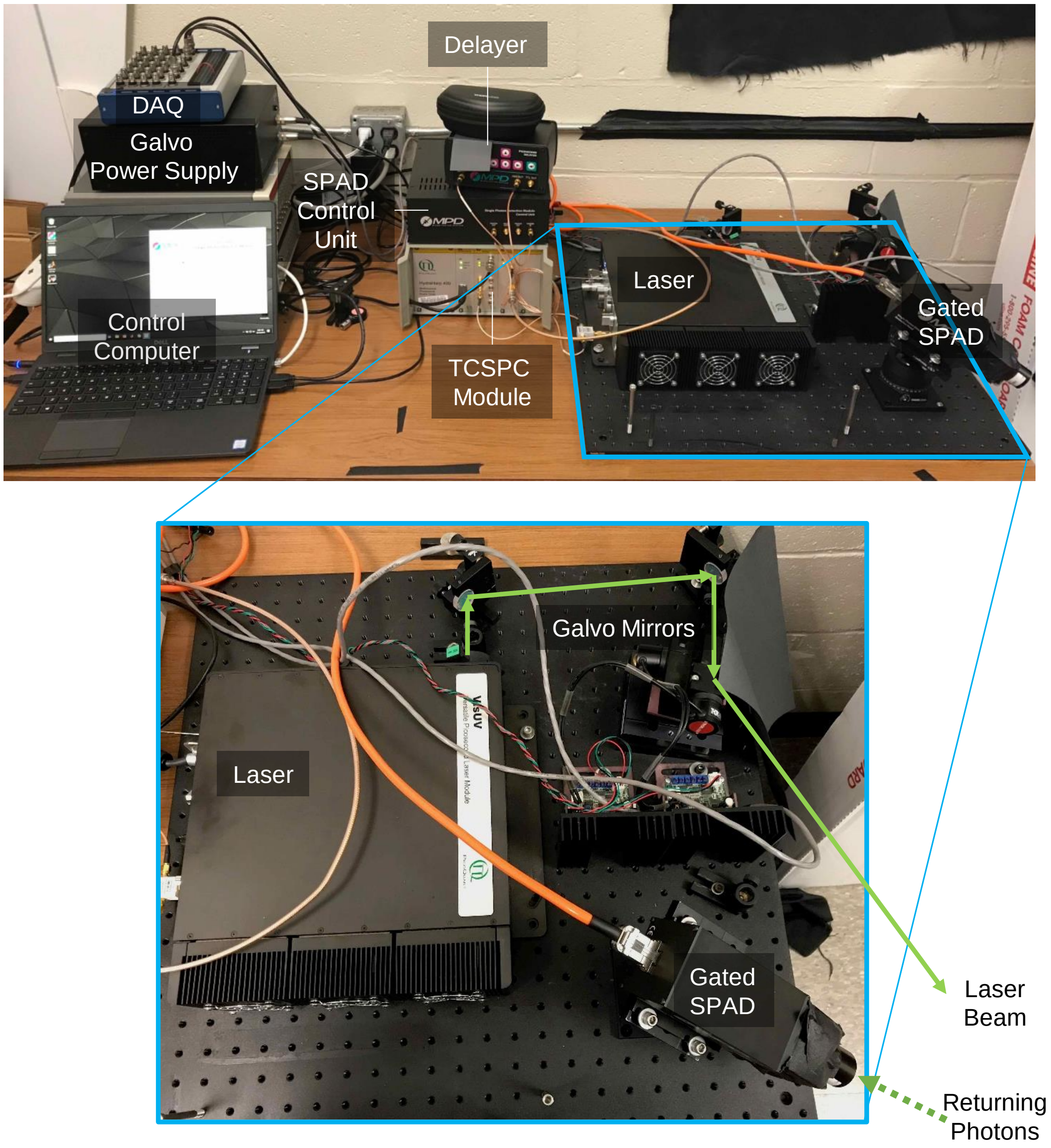

Supplementary Figure 8: Equipment setup used for the data acquisition. See Methods for a detailed description of the acquisition procedure.

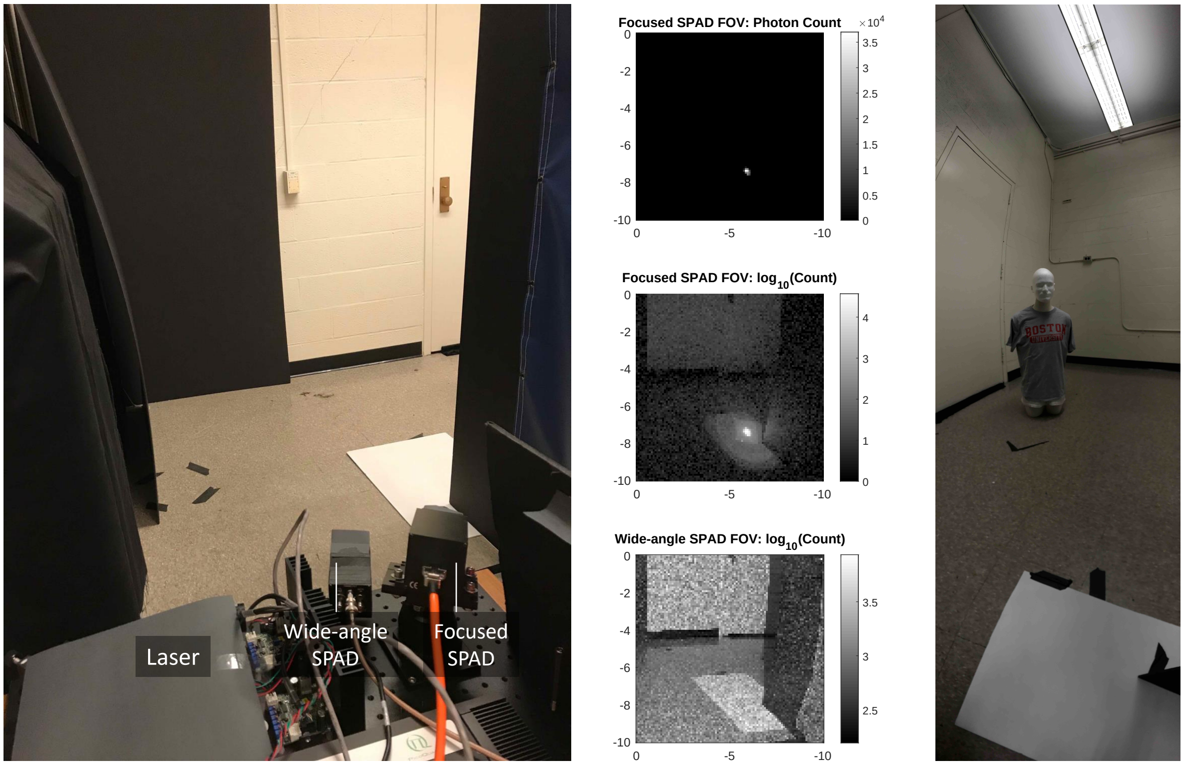

(a) (b) (c)

Supplementary Figure 9: Additional acquisition details. (a) Scene visible from the acquisition equipment. The nearby walls in the visible scene are darkened to reduce the noise from the visible scene from limiting the ability to reconstruct the hidden scene. (b) The SPAD is focused onto a small spot approximately 20 cm beyond the vertical edge. In order to determine the SPAD FOV, the laser is scanned over a large area, and the number of returned photons is recorded for 10 ms at each position. A large number of photons is recorded only when the laser is aimed within the SPAD FOV. Photon counts from laser positions outside the focused SPAD FOV are due to light that has undergone multiple reflections. A SPAD with a wide FOV can be used simultaneously to capture a more typical LOS image for reference. (c) An extended view of the hidden scene. Note that the reconstruction method performs well despite the ceiling lamp and the slight glossiness of the walls not being included in the model.

Supplementary Note 5: Example Data Set

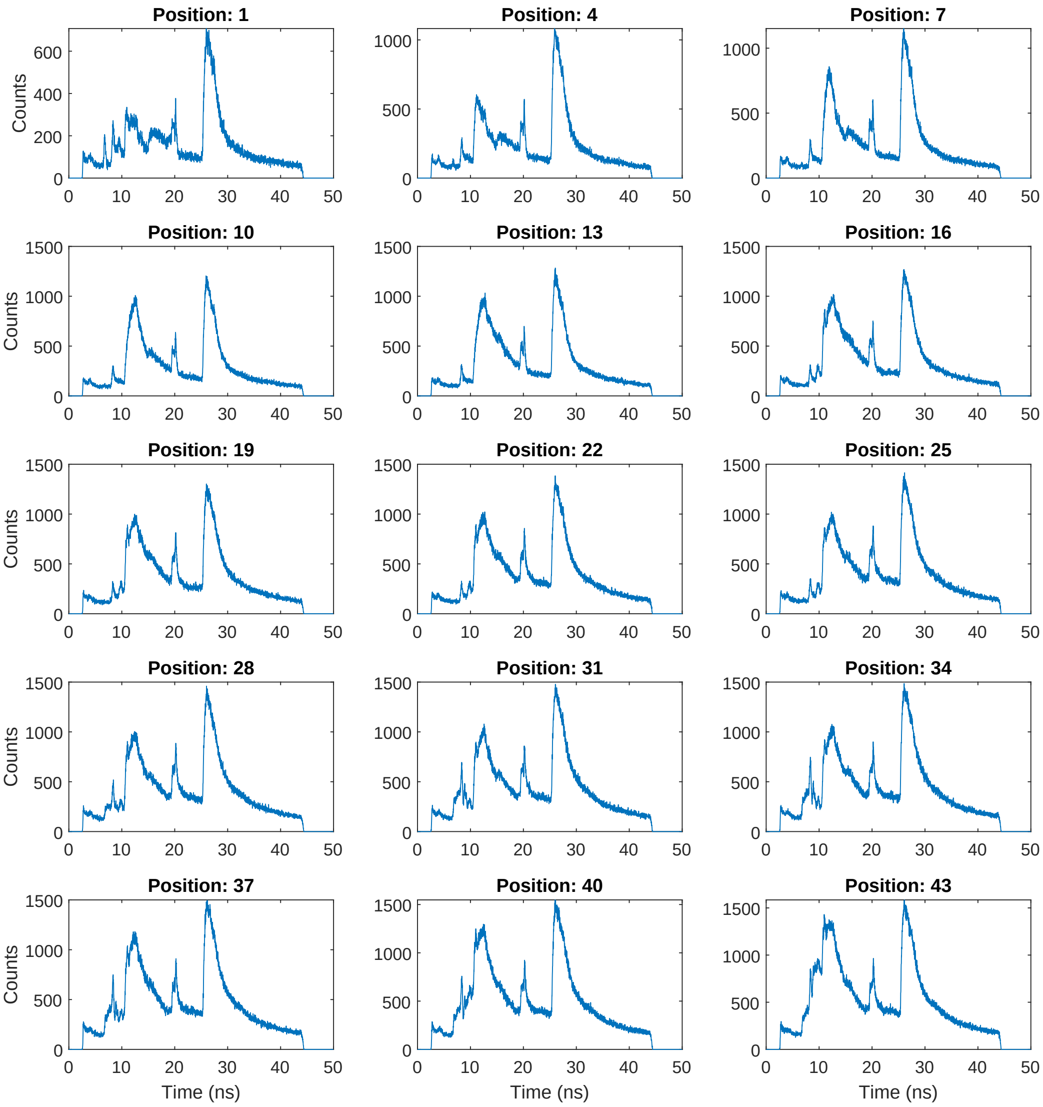

Supplementary Figure 10: A subset of the measured data for the Mannequins (20 secs) scene in the first row of Figure 4 of the main paper. Position 1 shows only the visible side contribution, whereas measurements from subsequent laser positions add contributions from the hidden scene.

Supplementary Figure 10 displays a subset of the directly measured photon detection time histograms used to form the reconstruction in the first row of Figure 4 (Mannequins, 20 secs). Supplementary Figure 11 shows a subset of the differences between sequentially measured histograms, corresponding to the light detected from individual wedges. Note that the histogram differences are far noisier than the histograms themselves. Included in the title of each subplot is the estimated *signal-to-clutter ratio* (SCR), which we define to be the ratio of the mean counts in histogram difference i to the mean counts in histogram i, containing all the counts from background, the visible scene, and previous wedges within the hidden scene. This value gives some indication of how much new information about wedge i is contained in histogram $i + 1$.

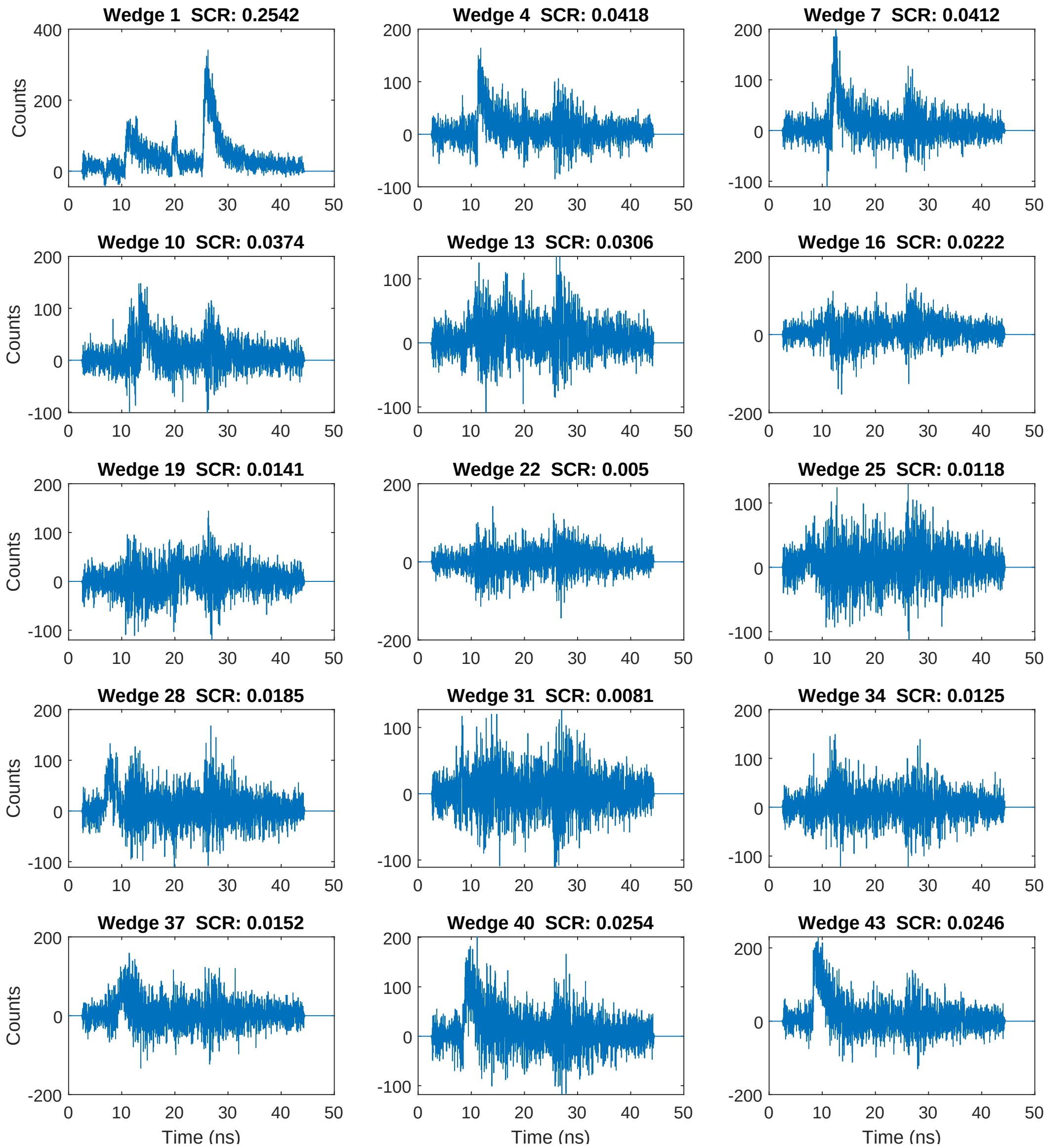

Supplementary Figure 11: A subset of the differences between sequentially measured histograms for the Mannequins (20 secs) scene in the first row of Figure 4 of the main paper. Signal-to-clutter ratio (SCR) refers to the ratio of the mean counts in the histogram difference for wedge i to the mean counts in the measurement histogram from position i.

Supplementary Note 6: Additional Results

Simulated Results

We performed additional simulations to investigate how ERTI behaves when the hidden scene does not exactly match our assumptions of having planar objects starting on the ground plane. The simulations shown here use a room of dimension 2.7 m × 2.9 m × 3.75 m, which is similar to the experimental test scenes. Measurements were computed for 49 measurements scanned along an arc of radius 1 cm.

The results for the empty room in Fig. 12(a) show good alignment of the wall positions and orientation, including sharp changes in orientation at the corners. We note that inter-reflections within the scene were not simulated, and are likely the reason that corners in the the experimental scenes are somewhat more rounded. Fig. 12(b) demonstrates that ERTI can handle the occlusion of the cylinder in front of the wall and also shows that the curvature of the cylinder is approximately matched by the changing orientation of facets in different wedges. The average height of the facets comprising the cylinder is within 0.04 m of the true height of 1.25 m. Fig. 12(c) and 12(d) show the effects of having a floating object – a planar

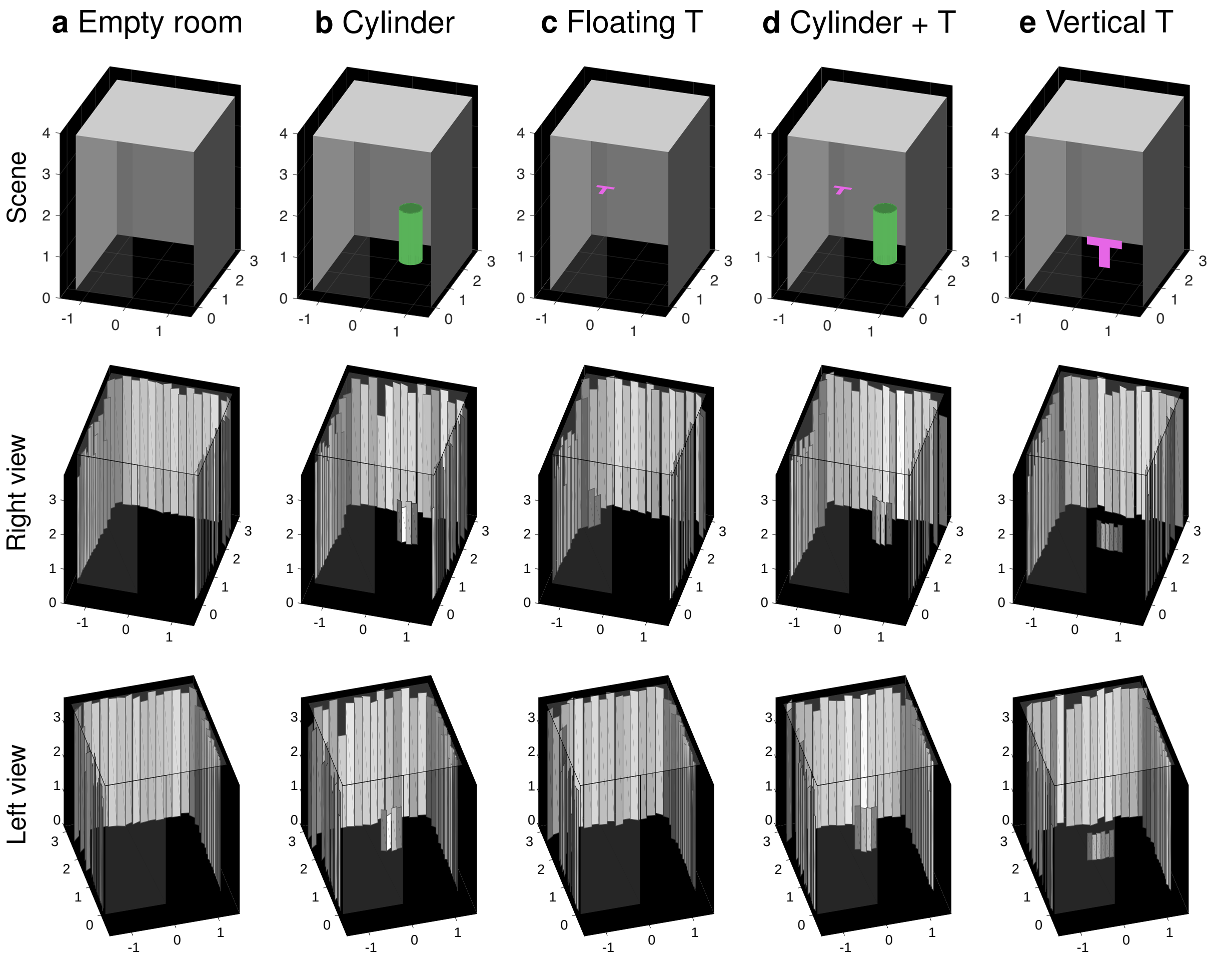

Supplementary Figure 12: Reconstructions for various simulated scenes show how ERTI handles curved surfaces, floating objects, and cantilevered shapes. Simulated scenes include (a) an empty room, (b) the same room with a cylinder (shown in green), (c) the room with a floating T shape (in pink), (d) both the cylinder and floating T shape, and (e) the room with a vertical T shape with cantilevered arms. The ERTI reconstructions recover only accurately the contents of each wedge connected to the floor.

T shape at 2.5 m – that does not match ERTI's gravity prior. Instead of being recovered as a floating object, we observe that instead, the T shape results in a few spurious planar facets located around 2.5 m from the vertical edge in the back-left corner of the room. However, these distortions of the true room shape are minor and limited to only the few wedges that actually contain the T shape. Finally, Fig. 12(e) shows the results of imaging an object – the vertical T shape – with cantilevered components. Like with the floating T, the reconstruction algorithm places the floating arms of the T at the correct radial distance within the wedge but enforces the requirement that facets be connected to the floor.

Additional Experiments

The results shown in Supplementary Figure 13 depict additional scenes acquired with the same general procedure described in the Methods, but with some some variations in the imaging subject, acquisition time per spot, and numbers of illumination spots. The top row shows the recovery of a scene from 37 illumination spots and with a challenging target: a mannequin wearing a red shirt that reflects very little of the green laser light. The middle row shows a large, empty room recovered from 73 illumination

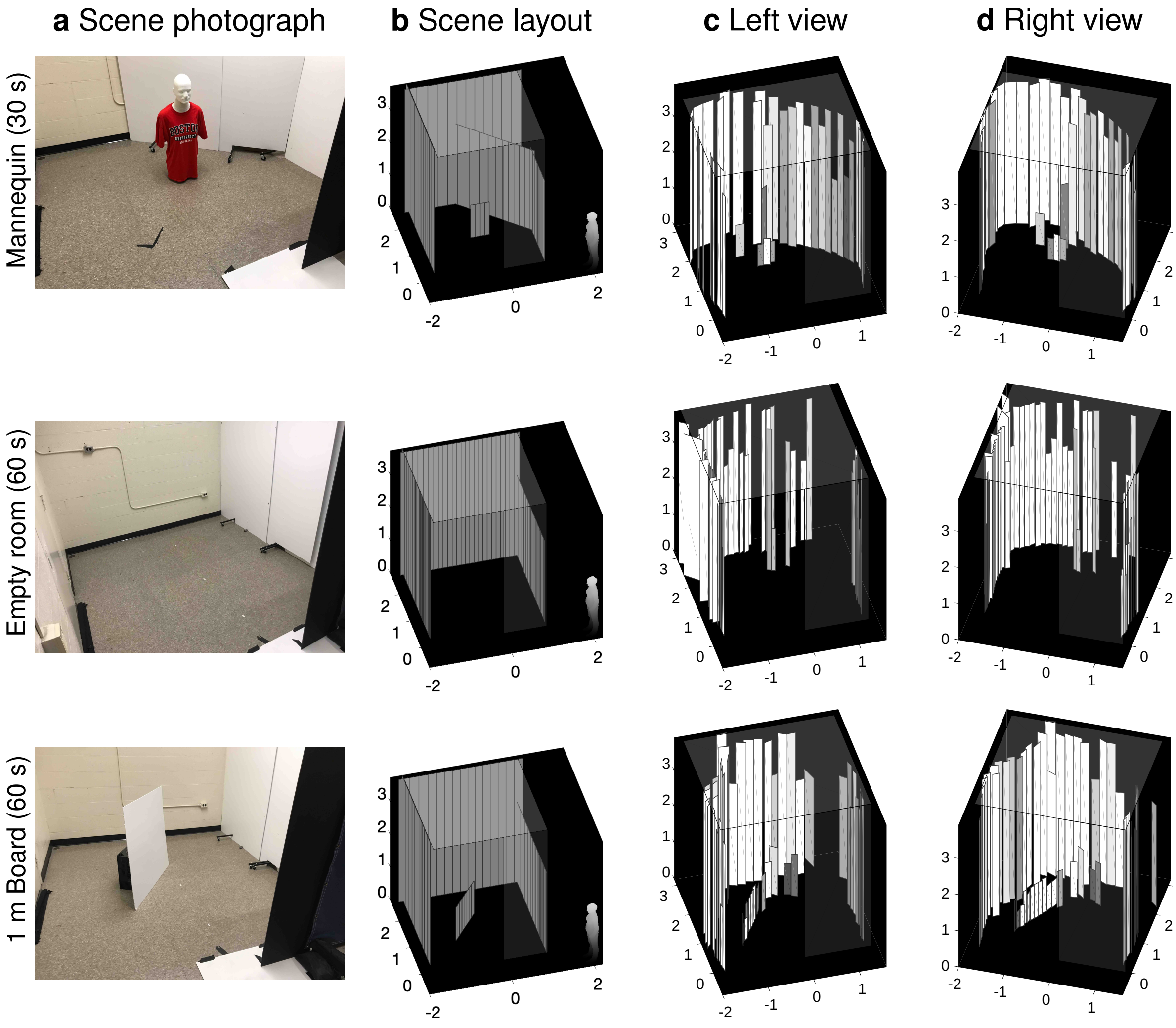

Supplementary Figure 13: Reconstructions from additional acquisitions reinforce the performance of the acquisition and algorithmic approaches.

positions. The bottom row recovers the correct angle, position, and height of a 1 m $\times$ 1 m square planar target from 45 illumination spots.

Effect of Acquisition Time

The experimental measurements were performed by time-tagging individual photons, so the histogram acquisition times could be post-selected as desired. In Supplementary Figure 14 we show results for the main results in the manuscript at acquisition times of 10, 20, 30, and 60 seconds per illumination spot. Recall that each of the acquisitions above used 45 illumination spots.

Analyzing the reconstructions for each scene for different times illustrates the performance of the ERTI reconstruction algorithm. We observe that reasonable reconstructions of the scene layout can be formed with as few as 10 seconds per spot (7.5 minutes for the whole scene). However, longer acquisition times generally lead to better accuracy, particularly in the facet height and orientation. For example, in the Mannequins scene, more of the partially-occluded wall facets are recovered with 60 seconds per spot. We do note that the reconstructions do not universally improve with longer acquisition times. Mismatch between the transient forward model and the experimental data become more apparent at higher acquisition times, so spurious facets are occasionally identified.

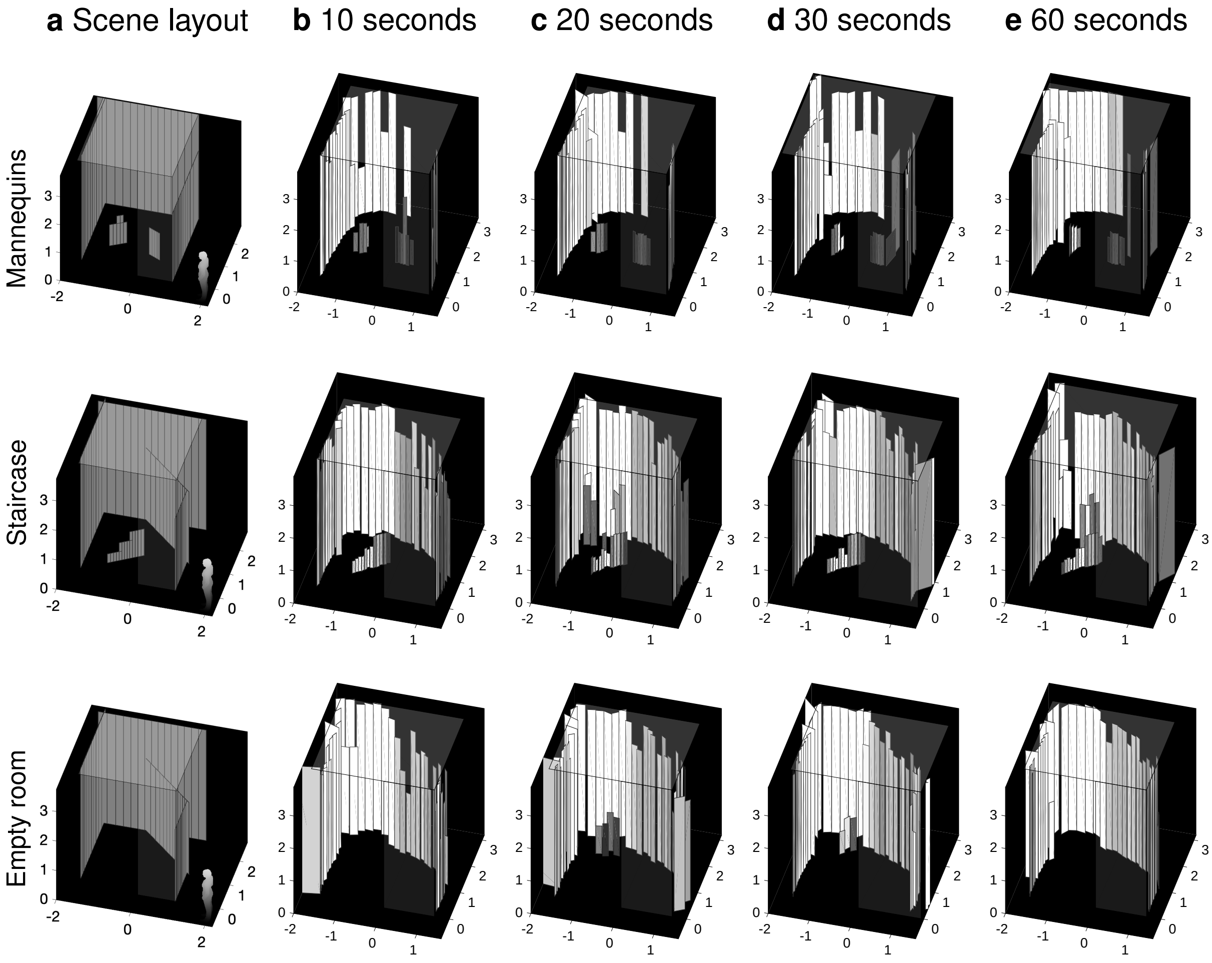

Supplementary Figure 14: Effects of varying acquisition time on ERTI reconstruction performance. Acquisition times shown are the measurement duration per illumination spot. Each of the acquisitions above used 45 illumination spots.

Supplementary Note 7: Robustness Analysis

Both ambient background light and visible scene contributions increase estimation difficulty. Although the mean contributions from both the background and visible scene cancel out due to the differencing of histograms, they still contribute to the variance of the measurements. Ambient background light contributions are assumed to be constant in time and so provide a constant background level within the measurement histograms. Visible scene light is time-varying and results in contributions similar to those of the visible scene, but that are approximately constant within each measurement histogram. This results in increased variance at different times within the measurements, making facets in the hidden scene, at distances similar to surfaces in the visible scene, more difficult to estimate. Similarly, the contributions from the ceiling, although modelled in our reconstruction, also contribute to the variance in a similar way, making estimation of the facets within the scene more challenging.

Supplementary Figure 15(a) shows simulated results for varying signal-to-background (SBR) ratios. SBR is defined as ratio of the total sum of signal photons from the hidden scene, to the total sum of background photons. A room with the same dimensions to that of the one in the main experimental results was simulated to generate ground truth measurement histograms. An appropriate, constant background level is added to each achieve the desired SBR (given an average photon count of 125 per time bin – similar to the experimental measurements). The reconstruction algorithm was run using this measurement data (25 trials with new Poisson noise realizations), and the number of facets with parameters correctly identified within tolerances is presented.

Supplementary Figure 15(b) shows similar results for varying signal strength. Signal strength here is defined by the average number of signal photon counts per time bin, given a time bin resolution of 16 ps and repetition rate of approximately 20 MHz. These were simulated with an SBR of 100.

Supplementary Figure 16 shows the percentage of facet distances estimated correctly within a tolerance

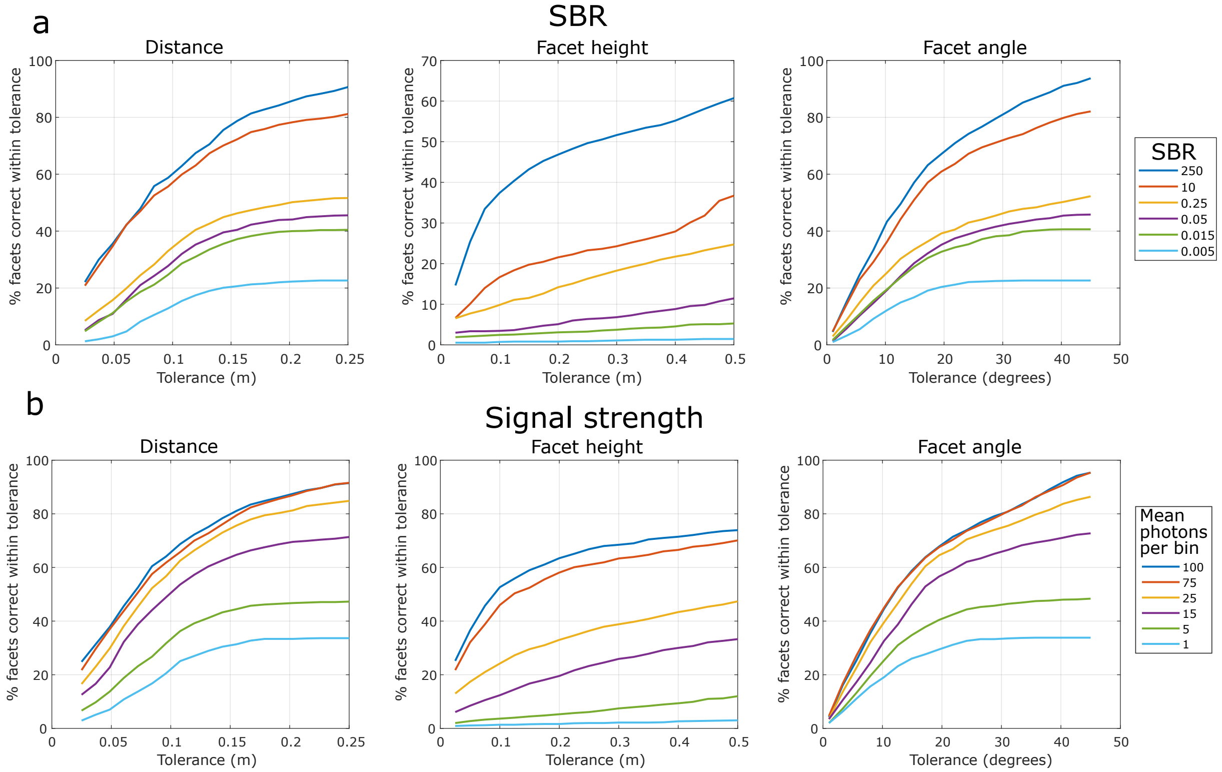

Supplementary Figure 15: Simulated robustness results. (a) Parameter estimation success at varying signal-to-background ratios. (b) Parameter estimation success with varying signal strength.

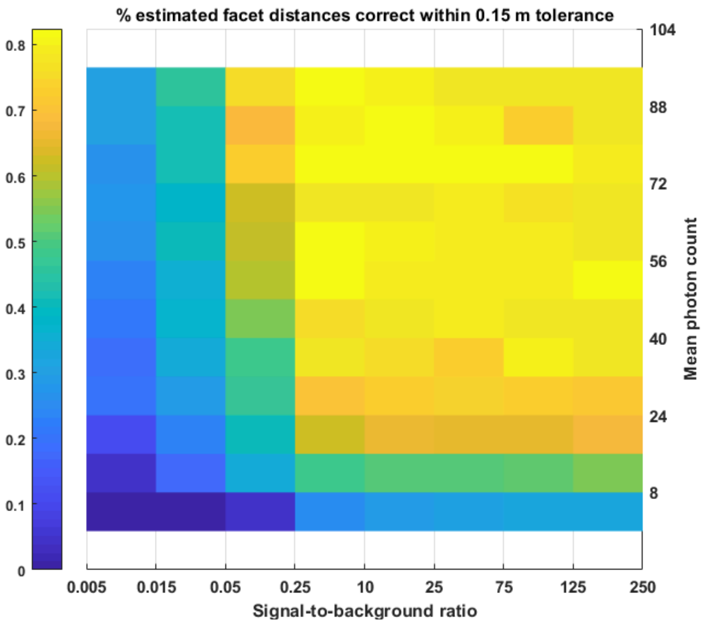

Supplementary Figure 16: The number of facet distances estimated correctly within a tolerance of 0.15 m, as a function of SBR and signal strength.

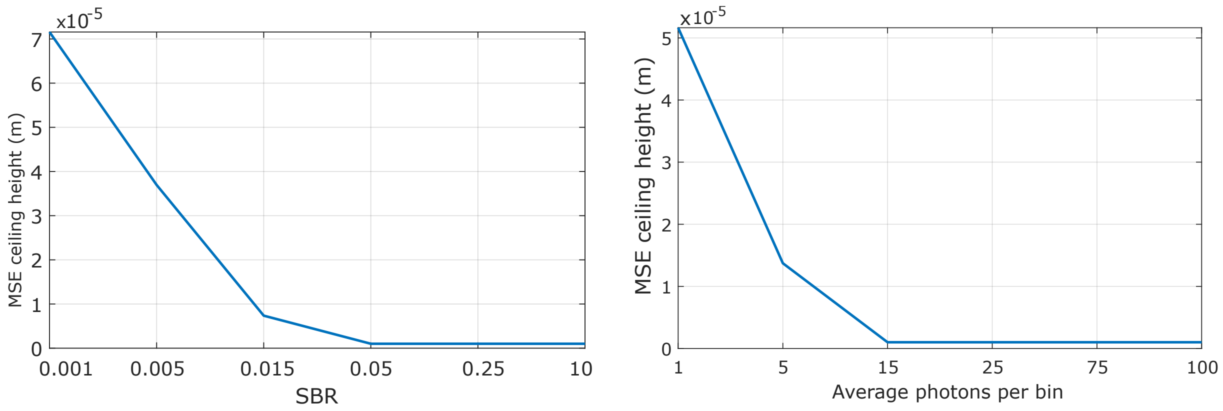

Supplementary Figure 17: Mean squared error for ceiling height estimation at varying SBR and signal strength.

of 0.15 m, as a function of both SBR and signal strength. This can be useful to identify regimes in which the the system can operate with success.

Supplementary Figure 17 shows simulated results for the mean squared error of ceiling height estimation, using the same methods. We see that the mean squared error in general is extremely low, suggesting we can get accurate ceiling height estimations at a wide range of signal strengths and background strengths. This is due to the fact the ceiling is a strong signal, present in every measurement histogram. Every histogram is used in the ceiling height estimation at once, so given N illumination positions, we have N observations of the ceiling we use to form the estimate.

Supplementary References

[1] Heide, F., Xiao, L., Heidrich, W. & Hullin, M. B. Diffuse mirrors: 3D reconstruction from diffuse indirect illumination using inexpensive time-of-flight sensors. In *Proc. IEEE Conf. Comput. Vis. Pattern Recognit.*, 3222–3229 (2014).

[2] Thrampoulidis, C. *et al.* Exploiting occlusion in non-line-of-sight active imaging. *IEEE Trans. Comput. Imag.* **4**, 419–431 (2018).

[3] O'Toole, M., Lindell, D. B. & Wetzstein, G. Confocal non-line-of-sight imaging based on the light-cone transform. *Nature* **555**, 338–341 (2018).

[4] Skellam, J. G. The frequency distribution of the difference between two Poisson variates belonging to different populations. *J. Royal Statistical Society* **109**, 296 (1946).

[5] Varin, C., Reid, N. & Firth, D. An overview of composite likelihood methods. *Statistica Sinica* **21**, 5–42 (2011).

[6] Tachella, J. *et al.* Bayesian 3D reconstruction of complex scenes from single-photon lidar data. *SIAM J. Imaging Sciences* **12**, 521–550 (2019).

[7] Brooks, S., Gelman, A., Jones, G. & Meng, X.-L. *Handbook of Markov Chain Monte Carlo* (CRC Press, 2011).

[8] Baddeley, A. J. & Van Lieshout, M. N. M. Area-interaction point processes. *Ann. Inst. Statistical Math.* **47**, 601–619 (1995).

[9] Rue, H. & Held, L. *Gaussian Markov Random Fields: Theory and Applications* (Chapman and Hall/CRC, 2005).
