# Supplementary material for: Seeing around corners with edge-resolved transient imaging
Source: Nat Commun. 2020 Nov 23;11:5929. doi: 10.1038/s41467-020-19727-4 (PMC7683558; doi:10.1038/s41467-020-19727-4)
Supplement: Supplementary file 2 — Description of Additional Supplementary Files [file 41467_2020_19727_MOESM2_ESM.docx]

**Description of Additional Supplementary Files**

**Supplementary Movie 1:**

**Description of edge-resolved transient imaging.** This movie provides a narrated description of the imaging setup, data acquisition procedure, response modeling, and reconstruction algorithm.
